# A method for reconstruction of interpretable brain networks from transient synchronization in resting-state BOLD fluctuations

**DOI:** 10.3389/fninf.2022.960607

**Published:** 2023-01-12

**Authors:** Yusuke Noro, Ruixiang Li, Teppei Matsui, Koji Jimura

**Affiliations:** ^1^Department of Biosciences and Informatics, Keio University, Yokohama, Japan; ^2^Department of Physiology, The University of Tokyo School of Medicine, Tokyo, Japan; ^3^Department of Biology, Okayama University, Okayama, Japan; ^4^PRESTO, Japan Science and Technology Agency, Tokyo, Japan; ^5^Department of Informatics, Gunma University, Maebashi, Japan

**Keywords:** resting-state fMRI, task fMRI, temporal dynamics, individual difference, Human Connectome Project

## Abstract

Resting-state (rs) fMRI has been widely used to examine brain-wide large-scale spatiotemporal architectures, known as resting-state networks (RSNs). Recent studies have focused on the temporally evolving characteristics of RSNs, but it is unclear what temporal characteristics are reflected in the networks. To address this issue, we devised a novel method for voxel-based visualization of spatiotemporal characteristics of rs-fMRI with a time scale of tens of seconds. We first extracted clusters of dominant activity-patterns using a region-of-interest approach and then used these temporal patterns of the clusters to obtain voxel-based activation patterns related to the clusters. We found that activation patterns related to the clusters temporally evolved with a characteristic temporal structure and showed mutual temporal alternations over minutes. The voxel-based representation allowed the decoding of activation patterns of the clusters in rs-fMRI using a meta-analysis of functional activations. The activation patterns of the clusters were correlated with behavioral measures. Taken together, our analysis highlights a novel approach to examine brain activity dynamics during rest.

## Introduction

Resting-state functional MRI (rs-fMRI) is a functional neuroimaging technique during which subjects are at rest and not engaged in any behavioral task, and it is thought to monitor intrinsic physiological signals in the brain (Biswal et al., 1995). Several studies have identified brain-wide large-scale spatiotemporal architectures as resting-state networks (RSNs), within a frequency of 0.1–0.01 Hz (Raichle et al., 2001; Fox et al., 2005, 2016; Damoiseaux et al., 2006; Fox and Raichle, 2007). Because of its technical ease, rs-fMRI has also been applied to patients with neuropsychiatric disorders and has revealed alterations of RSNs related to these disorders (Buckner et al., 2008; Du et al., 2016).

RSNs are usually investigated by examining temporal correlations of resting-state activity, also known as resting-state functional connectivity (RSFC), in multiple brain areas comprising the network. Although prior studies assessed RSFC in RSNs using the entire duration of rs-fMRI scans (static RSFC), recent studies have examined fluctuations in RSFC for timescales ranging from seconds to a few minutes, so-called time-resolved RSFC (Chang and Glover, 2010; Sakoglu et al., 2010; Handwerker et al., 2012; Jones et al., 2012; Hutchison et al., 2013; Lindquist et al., 2014; Zalesky et al., 2014). One important characteristic of time-resolved RSFC is its temporal fluctuation over a large range of correlation values (Zalesky et al., 2014; Betzel et al., 2016). Some studies classified time-resolved RSFC into distinct clusters that temporally evolve during rs-fMRI scanning (Leonardi et al., 2013; Allen et al., 2014). These clusters of time-resolved RSFC have been associated with structural connectivity (Hansen et al., 2015), aging (Faghiri et al., 2018), and mental disorders (Damaraju et al., 2014; Rashid et al., 2014; Su et al., 2016). Thus, the spatiotemporal dynamics of rs-fMRI activity may provide more information about the brain and phenotype than static RSFC.

**Figure 1:**
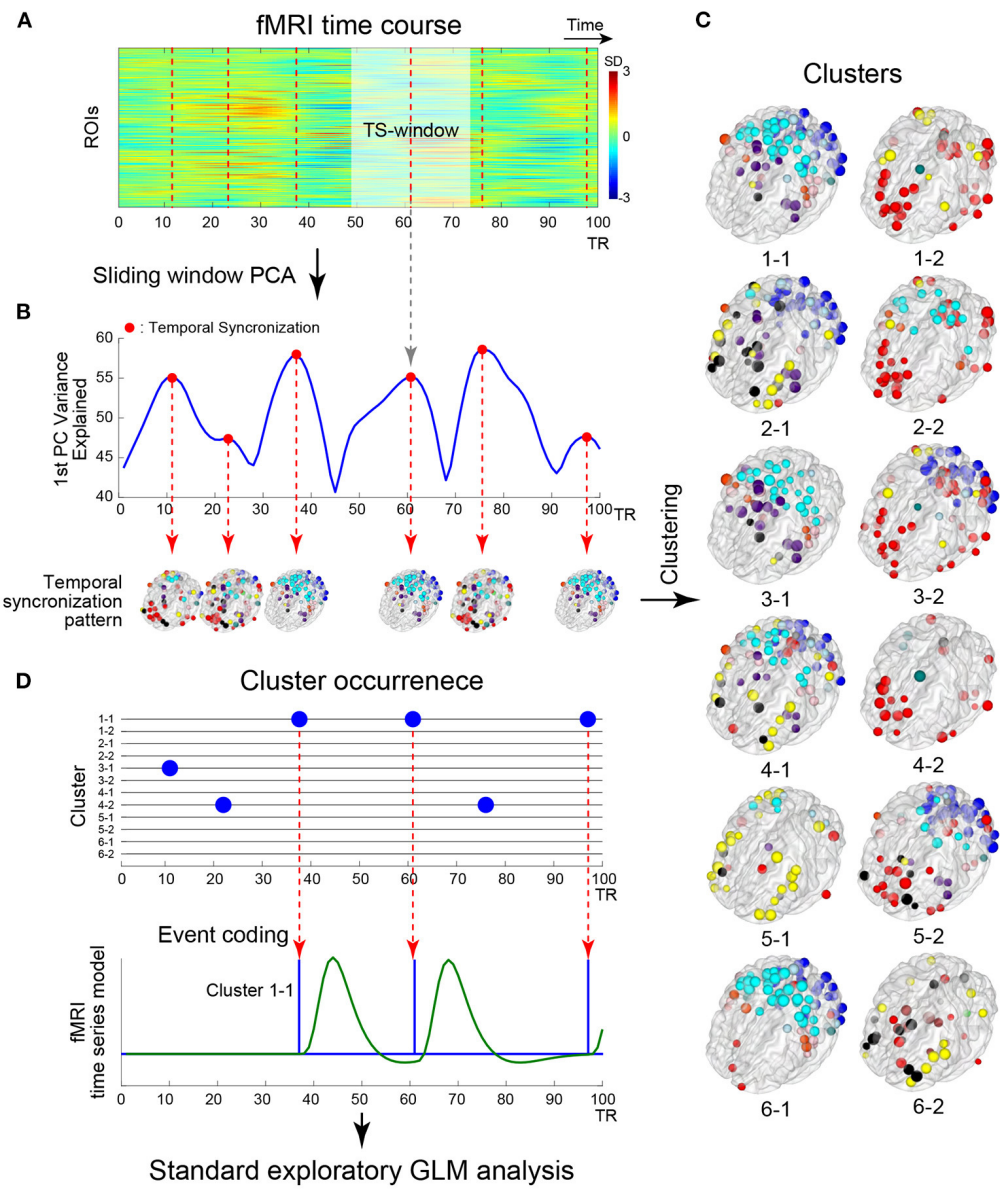
Schematic illustration of the analysis framework. **(A)** Example timecourses of fMRI signals extracted from regions of interest (ROIs). The vertical and horizontal axes indicate ROIs and time, respectively. The color indicates the magnitude of standardized fMRI signals. The brighter rectangle area indicates a window where ROIs timecourses showed temporal sectorization [TS; see **(B)**], and broken vertical red lines indicate TS time points. **(B)** Example timecourse of the variance explained by the first principal component (1^st^ PC, blue line). Red dots indicate local maxima of the variance, when fMRI signal temporally synchronized. ROIs showing strong synchronization were mapped onto the 3D surface of the brain. Colors of the ROIs indicate resting networks identified in prior work (Power et al., 2011). **(C)** Temporal synchronization patterns were labeled as clusters based on a transition matrix across the cluster in Supplementary Figure S3A. ROI-based pattern for each 12 cluster centroids comprising 6 pairs of clusters mapped onto the 3D surface of the standard brain. **(D)** Timecourses of cluster occurrence (top). The vertical and horizontal axes indicate clusters and time, respectively. The occurrences of clusters are indicated by blue dots. The occurrences of the clusters were coded as events in fMRI time series modeling (bottom). Blue and green solid lines indicate event onsets and BOLD model of the cluster occurrence. Whole-brain exploratory standard event-related GLM analysis was performed using resting-state data.

To provide an intuitive and interpretable characterization of the spatiotemporal dynamics of rs-fMRI, herein we developed a new method to extract dominant patterns in rs-fMRI activity and relate these patterns to well-known task-evoked activation patterns at voxel-based resolution. To examine time-resolved RSFC, we used a sliding-window approach with a short time window (Figure 1A). Unlike many previous studies of time-resolved RSFC that required large sliding-windows (<100 s) to examine detailed patterns of RSFC (Allen et al., 2014; Leonardi and Van De Ville, 2015; Zalesky and Breakspear, 2015), we used a smaller sliding window and focused on detecting dominant patterns of RSFC that reflected transient brain-wide high connectivity (Zalesky et al., 2014; Betzel et al., 2016; Vohryzek et al., 2020; Figure 1B). We first used region-of-interest (ROI) analysis to classify the spatiotemporal patterns emerging in transient high connectivity into distinct clusters (Figure 1C). We then reconstructed the voxel-based patterns associated with the clusters using linear regression (Figure 1D). Voxel-based visualization allowed us to compare the activation patterns of the clusters and task-related functional maps and then examine individual differences in the activation patterns of the clusters using correlations with behavioral and psychological measures.

The novelty of the proposed method is that it simultaneously enabled detection of neural activity events at high signal-to-noise ratio and visualization of activity the patterns at the voxel level. Previous studies such as the co-activation pattern analysis (Liu and Duyn, 2013) conducted event detection with voxel-level resting state fMRI data. However, because single voxel timecourses are noisy, event detection using the voxel-based data would be noisy. To address this problem, the proposed method used event detection with region-of-interest (ROI)-based data and principal component analysis (PCA). However, PCs obtained with ROI-based data are spatially compressed and cannot be directly compared to voxel-based activity maps. Therefore, following the event detection, the proposed method conducted a generalized linear model (GLM) analysis based on extracted events in voxel-based data to obtain voxel-level description of whole-brain activity-patterns associated with the events.

## Materials and methods

### Datasets

Resting-state and task-related fMRI data were obtained from the Human Connectome Project (HCP: http://www.humanconnetome.org/; Glasser et al., 2016; *n* = 810), with all data included in the S900 data release. For each subject, resting-state scans (approximately 15 min) were conducted for four functional runs with a repetition time (TR) of 0.72 s and 2-mm isotropic spatial resolution.

### Image preprocessing

rs-fMRI data were preprocessed by HCP and cleaned by ICA-based X-noiseifier (FIX) from the FMRIB Software Library (FSL) (Griffanti et al., 2014; Salimi-Khorshidi et al., 2014). The current study did not apply global signal regression. Task fMRI data were also preprocessed with a procedure identical to that for resting-state fMRI data, including FIX cleaning. MRI signal timecourses were extracted from 264 ROIs distributed across the whole brain identified in prior studies (Power et al., 2011). We have selected this ROI atlas because it is one of the most frequently used ones to examine brain-wide functional networks in humans (Keerativittayayut et al., 2018; Matsui et al., 2022a), thus allowing us to compare present results directly with other literatures.

A temporal bandpass filter (0.009 <f <0.08 Hz) was applied (Power et al., 2014), and then scanning frames of the first and last 38 TRs in each functional run were discarded from the analysis. This resulted in 264 ROIs × 1,124 time points.

### Time-resolved analysis

The analysis was based on the framework illustrated in Figure 1. Because we focused on brain-wide high synchronization (Zalesky et al., 2014; Betzel et al., 2016; Figure 1B), we applied a sliding window principal components analysis (sw-PCA) (Vohryzek et al., 2020; Figure 1A). PCA was applied to fMRI timecourses in ROIs in a window (25 TRs =18 s) that slid over the entire timecourse, providing 1,100 principal components (PCs) along a temporal axis with 264 ROIs × 25 time points across 1,124 (1,200 – 38 × 2) full time points (Figure 1A; see below for the timecourses with different window sizes). The fMRI timecourses were z-normalized in each window to examine the relative change in the window, so that (1) the PCs of normalized timecourses were equal to eigenvectors of the correlation matrix of timecourses within the window and (2) the explained variance was identical to eigenvalues of the correlation matrix. Temporal synchronization (TS) was defined as time windows where the timecourse of the 1^st^ PC's explained variance was at local maxima (Figure 1B). Thus, TS indicates time points in which fMRI timecourses are locally and transiently synchronized (Figure 1B; Allen et al., 2014). To examine the consistency of results between the current study and prior studies, the following synchronization measures were also calculated: global efficiency (Zalesky et al., 2014), variance of correlation (Allen et al., 2014), phase synchronization approach (Ponce-Alvarez et al., 2015; Senden et al., 2017) and epoch numbers at frames as point-process analysis (PPA) approach (Tagliazucchi et al., 2012; Liu and Duyn, 2013; Supplementary Figure S2).

A temporal synchronization pattern (TSP) was then defined as the 1^st^ PC of sw-PCA when a TS occurred (Figure 1B). Because the sign of PC is not determined uniquely, for each window, we adjusted the sign of the 1^st^ PC such that it showed a monotonical increase toward the TS time point (Supplementary Figure S1C). The current study adopted the 1^st^ PC, instead of a correlation pattern defined as a correlation matrix of partial fMRI timecourse in a window (e.g., Allen et al., 2014). This is because the current study used a short window size, which makes the correlation matrix estimation unstable (Leonardi and Van De Ville, 2015). Thus, using the 1^st^ PC, we focused on an overall pattern of correlation in a window with TS.

### Exploration of window size

The size of the sliding window is a critical parameter in time-resolved RSFC analyses, because an excessively short window introduces spurious fluctuations (i.e., increasing the false positive rate; Leonardi and Van De Ville, 2015; Zalesky and Breakspear, 2015), whereas a longer window tends to reduce the sensitivity to high frequency temporal dynamics. To explore the effect of window size, we calculated the variance explained by the 1^st^ PC of windowed timecourses for window sizes ranging from 15 to 65 TRs. Supplementary Figure S1 shows the results for a participant (ID: 105115). Within the range of 15–35 TRs, TSP timecourses temporarily fluctuated, suggesting that phase-synchronized signals were overall increasing or decreasing around the local maxima. Additionally, within this range, local maxima of the explained variance were almost consistent, indicating that the center time point of a TS window is almost independent of window size.

However, with window size >45 TRs, the timecourses contained many small-peaked maxima and were not temporally aligned. With long window sizes, it was difficult to detect some of the local maxima observed with shorter window sizes. Although multiple PCs based on a correlation matrix (Allen et al., 2014) is effective for such larger window sizes, use of multiple PCs was not applicable to the current TS analysis due to the procedure of sign flipping for PCs. Taken together, the current study used 25 TRs for the window size to indicate the overall change of signals in synchronization.

### Reducing motion-related artifacts

To minimize motion-derived artifacts against TS patterns, framewise displacement (FD) from derivatives of the six rigid-body realignment parameters were calculated over the scanning run (Power et al., 2012). If a large head movement occurred (FD > 0.5), to ensure that the head movement did not affect data in a window (Power et al., 2017), we discarded TS window from the head movement to 30 TR after the head movement. Additionally, subjects with 60 TS in any session were discarded from analyses. Because prior studies used more stringent criteria to discard scanning frames (FD > 0.2), we examined the probability of head movements with FD > 0.2 within TS windows.

### Clustering of temporal synchronization patterns (TSPs)

To evaluate similarities among TSPs, we used k-means clustering with the number of clusters set to k = 12 for TSPs from all subjects and all sessions (approximately 200,000 TSPs in total; Figure 1C). The distance between two TSPs was defined as pair-wise spatial correlations between TSPs. The k-means clustering was repeated 100 times with random initialization and, over the repetition, the result with the minimum summation of distances from TSP to cluster centroids was employed. Then, the center time point of a TSP window was labeled by the number indicating the cluster (Figures 1C, D).

### TSP-related whole-brain mappings

**Figure 2:**
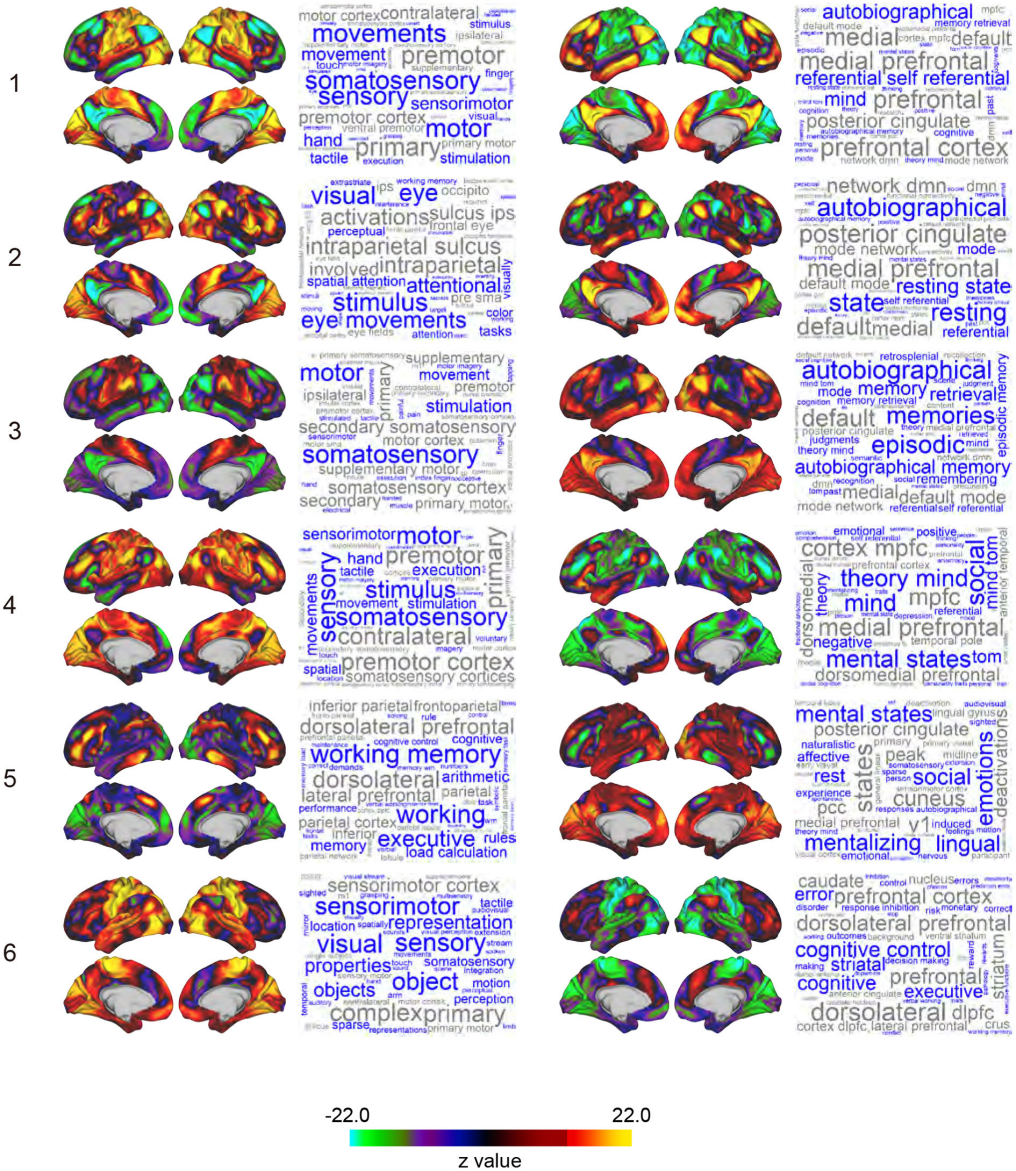
Whole-brain maps of SAP. Z-values of signal magnitude were color-coded and mapped onto the 3D surface of the standard brain. Columns and rows indicate clusters (1-6) and pairs of sub-clusters (e.g., Sub-clusters 1-1 and 1-2). Each inset shows **left** lateral, **right** lateral, **left** medial, and **right** medial surfaces of the brain. Each SAP map is functionally characterized by a word cloud on the **right** based on a map-to-function decoder. The font size of a word represents the strength of similarity between the SAP maps and functional brain maps revealed by meta-analyses. Names of brain regions are colored in gray.

Because TSPs were defined at ROI-level, we developed a method to translate TSPs into voxel-based maps for easier interpretation. To obtain a voxel-based map related to ROI-based TSP clusters, we conducted a whole-brain exploratory analysis based on a standard event-related fMRI approach, whose events were time-locked to the TS windows (Figure 1D). This event coding was possible because PC timecourses were sign-adjusted such that they monotonically increased within TS windows (Supplementary Figure S1C). The TS events were defined as the centers of TS windows for each cluster label (Figures 1A, D) and were convolved with the canonical hemodynamic response function in FSL. Then, for each scanning run, parameters were estimated using a generalized linear model (GLM) by film_gls implemented in FSL suite (https://fsl.fmrib.ox.ac.uk/). Voxel-based data were expressed in grayordinates space (Glasser et al., 2013), which consisted of 91,282 voxels × 1,100 time points (i.e., a pixel-based analysis; Figure 2). For comparison, we conducted ROI-based analysis using the same procedure with 264 ROIs × 1,100 time points. The first and last 50 TRs in each scanning run were discarded from the analysis. Voxel- and ROI-based whole-brain synchronized activation patterns (SAPs) were constructed based on parameter estimates for each of the identified 12 TSP clusters (Figure 1C). The SAP maps were collected from all scanning runs and subjects, and group-level statistics were calculated based on a mixed-effect GLM implemented in FEAT in FSL, treating a scanning run as a fixed effect and subjects as a random effect. For pixel-wise analysis, group-level statistical maps were visualized using the Connectome Workbench platform (Marcus et al., 2013). For ROI-based analysis, ROIs with beta-values > 0 were mapped on a transparent 3D standard space based on BranNet Viewer (Xia et al., 2013).

The 12 SAPs could be grouped into 6 pairs of clusters that had anti-correlated spatial patterns and almost always co-occurred. These pair-wise anti-correlations can be explained by (1) the arbitrariness of the sign of PCs, (2) the spatial pattern of clusters, and (3) autocorrelation of BOLD timecourses. Therefore, we named these 6 groups as clusters and each SAP in a cluster as a sub-cluster. For example, Cluster 1 consists of Sub-clusters 1-1 and 1-2, and Cluster 2 consists of Sub-clusters 2-1 and 2-2. Note that we avoided the use of terms such as states and attractors because similar SAPs were obtained in phase-randomized null models (see Discussion for an interpretation of clusters).

### Characterization of SAP maps

To characterize the SAP maps, we decoded SAP maps based on their topographical structure (Margulies et al., 2016; Figure 2). Voxel-based reconstruction of SAP maps allowed us to use existing meta-analyses of functional brain mapping (https://neurosynth.org; Yarkoni et al., 2011; Gonzalez-Castillo et al., 2019; Matsui et al., 2022b) to decode weighted word lists that characterize the SAP maps. Large weights associated with the decoded words reflect greater topographical similarity between an SAP map and functional brain maps associated with the words in the meta-analysis database (Yarkoni et al., 2011). The word lists were visualized as a word cloud where the size of the words reflected the weights (Chen et al., 2018). Words unrelated to brain functions were eliminated.

### Analysis for temporal structure of relationships among cluster occurrences

To examine the temporal structure of cluster (sub-cluster) occurrences, occurrence probabilities were calculated based on the frequency of occurrence across time series. The probability was calculated for each of the 6 clusters. For each cluster, the probability associated with sub-clusters (e.g., 1-1 and 1-2) was averaged. Then, we examined point-wise mutual information (PMI; Church and Hanks, 1989) as a function of two time points: *t* and *t* + τ (*t*, τ > 0). PMI was calculated as the ratio of two observation probabilities (*X, Y*) of TS events of Clusters (*a, b*):


(1) $$\begin{array}{l} PMI\left(X_{a}=1,Y_{b}=1\right)=\log_{2}\left(\frac{\mathrm{Pr}\left(Y_{b}=1|\;X_{a}=1\right)}{\mathrm{Pr}\left(X_{a}=1\right)}\;\right), \end{array}$$

where, *X*_*a*_ = 1, if an event where Cluster *a* was observed at time *t* given a TS of any clusters occurring at time *t*, otherwise *X*_*a*_ = 0; and *Y*_*b*_ = 1, if an event where Cluster *b* was observed at time *t* + τ given the TSs of any clusters occurring at time *t* and t + τ, otherwise *Y*_*b*_ = 0.

The two probabilities were computed by counting observations for each pair at times *t* and *t* + τ. The timecourses of the probabilities were temporally smoothed using a moving average with window size = 50 TRs to avoid the denominator Pr(*X*_*a*_ = 1) reaching zero. Then, at each scanning frame, PMIs were calculated for all possible pairs of the 6 clusters.

Each PMI timecourse was used to quantify two temporal characteristics, (1) time effect reflecting monotonic change in PMI over the scanning period and (2) lag-time effect reflecting PMI change at time t and (*t* + τ), by using multiple regression analysis. PMIs in the first and last 100 time points were discarded from the analysis. The lag effect was examined for τ > 100 TR to minimize bias derived from autocorrelation of PMIs (Figure 4A). The time effect and lag effects were color-coded in the heat maps. PMIs without lag (τ = 0) were also estimated in the regression analysis.

### Evaluation of temporal structure

To evaluate the temporal characteristics of TSP, null data sets were generated by phase randomization of the real data for each session (Hindriks et al., 2016). Phase randomized null data preserve covariance and autocorrelation of timecourses (Liegeois et al., 2017). We used the phase-randomized null data set to test the hypothesis that clusters involve temporal relationships beyond autocorrelation of the BOLD timecourse. Results among subjects and sessions for the null dataset kept variabilities among subjects and sessions because the null time series were generated for each session of each subject separately.

### Relationships of TSPs during rest and tasks

To characterize the clusters identified based on TSPs, we analyzed TSPs during performance of 7 behavioral tasks of HCP (Barch et al., 2013). Scanning data were collected using a temporal and spatial resolution identical to the ones used during the resting state. TSPs during task performance were calculated in a similar way as for resting-state data (see above). Briefly, classification of task TSPs was performed by k-nearest neighbor classification (k = 100), and classification scores lower than the 5-percentile of the score of a random pattern were replaced with 0. Task-related activation was estimated based on the standard GLM procedure used in the HCP pipeline, and activation contrast maps were created for 23 onsets during 7 tasks (see Figure 5B for task contrast lists).

Then, relationships between TSP maps in resting state (Figure 2) and task-activation maps were examined by evaluating the inter-subject consistency between the two types of maps. The mean effects of SAP and of task activation across subjects were subtracted out from the TSP maps of individual subjects. Consistency was quantified as the spatial correlation between SAPs and task-related activation maps for each subject. To test the significance of spatial correlations, we compared two distributions of the correlations of SAP and task-related activation maps from identical subjects (tested correlation) and from different subjects (null distribution) and then calculated Hedges' g between the tested and null distributions (Supplementary Figure S7) using MATLAB toolbox “Measures of Effect Size” (Hentschke and Stüttgen, 2011; https://github.com/hhentschke/measures-of-effect-size-toolbox).

### Relationships between clusters and RSFC

Clusters were examined in relation to the RSFC, which was calculated as pair-wise correlation coefficients based on the full timecourse of the rs-fMRI scan (Fox et al., 2005; Power et al., 2011; Smith et al., 2015). For each subject, timecourses of 13 RSNs (Power et al., 2011) were extracted from the rs-fMRI runs and averaged across regions within each of the networks. Then, the strengths of RSFC between the networks were calculated as pair-wise correlation coefficients. RSFC strengths were predicted by group-level multiple regression analysis using the occurrence frequency of the clusters as predictors. R-squared values were collected from all subjects and averaged across subjects for each RSFC strength.

A similar multiple regression analysis was performed based on reconstructed maps of the clusters (Figure 2). For each subject and network, beta-values of the reconstructed maps were extracted and submitted to a multiple regression model using the RSFC and beta-values of the maps as predicted and predicting variables, respectively. Then, R-squared values were calculated as above. A regression analysis was performed with the beta-value of each cluster map as a factor.

### Relationships between TSPs and behavioral measures

To characterize TSPs in relation to psychological and behavioral characteristics, we explored cross-subject correlations between TSPs and behavioral measures. The HCP dataset includes behavioral measures [subject measures (SMs)] of individual subjects, including demographic, psychological, and behavioral measures, collected by standard procedures conducted outside the scanner. Detailed descriptions of SMs are available elsewhere (https://wiki.humanconnectome.org).

Prior studies found relationships between SM correlation mode and RSFC during resting state in the HCP dataset (Smith et al., 2015; Miller et al., 2016) by using canonical correlation analysis (CCA; Hotelling, 1936). Based on these studies, we also used CCA to find correlations between RSFC and selected SMs. The adoption policy of SMs in the current study was similar to that in a prior study (Smith et al., 2015). More specifically, we excluded the following SMs: (1) those with insufficient variability with 95% of subjects rated by the same measure value, (2) those treated as confounds, (3) those correlated with other measures, (4) those that were undesirable to feed into the CCA analysis or had high correlation with already-selected measures (Smith et al., 2015), (5) family history measures, (6) and age adjusted measures. The following measures of behavioral performance during task fMRI were added: accuracy of (1) 2-back working memory task, (2) resemble task, (3) social cognition task, (4) story block in language task, and (5) math block in language task. The total number of SMs used in the analysis was 106.

Prior to CCA analysis, images and SMs were preprocessed. First, for each subject, two SAP maps corresponding to two sub-clusters in a cluster (e.g., Sub-cluster 1-1 and 1-2) were concatenated, resulting in 6 images each involving 182,564 pixels (91,282 pixels × 2 sub-clusters). The images were subjected to rank-based inverse Gaussian transformation to avoid the influence of potential outlier values (Van der Waerden, 1952). Second, the effects of potentially confounding variables were subtracted out from SMs before calculation of SAP and cluster occurrence using multiple regression analysis. The confounding variables included age, sex, age^2^, age^2^ × sex, height, weight, acquisition reconstruction software version, and FD averaged in the scanning session. Finally, to avoid over fitting, PCA was applied to SMs and the 6 images prior to CCA. The number of dimensions in PCA was set to 50 because 50 PCAs explained more than 90 and 18% of SM variances and image variances, respectively. In a separate control analysis, the dimension of the PCA was increased to 100, and CCA was applied similarly. We confirmed a consistent CCA mode (Supplementary Table S1).

CCA statistics were tested for significance by using random permutation of subjects (10,000 permutations). *P*-values were corrected for multiple comparisons based on family-wise error rate. Finally, SMs and SAP weights were calculated as correlation coefficient across subjects between CCA mode and SMs/SAP. To examine the relationships between cluster occurrences and SMs, a pairwise correlation coefficient was calculated for each cluster.

## Results

### Extraction of transient spatiotemporal features

Figure 1 illustrates the analysis framework of the current study. From the publicly available HCP dataset, rs-fMRI timecourses were extracted for ROIs across the whole brain (Power et al., 2011; Figure 1A). To examine the temporal characteristics of rs-fMRI signals, we used a sliding window approach, which is a simple but powerful tool to examine fluctuations of brain-wide high and low correlations of activities during resting state (Zalesky et al., 2014; Betzel et al., 2016).

We first examined high correlation periods based on a classification of spatiotemporal activation patterns. Instead of calculating temporal correlations in the sliding window, ROI timecourses were submitted to sw-PCA (Vohryzek et al., 2020). The 1^st^ PC explained the largest fraction of variance of resting-state brain activity within a window. Timecourses of the variance explained by the 1^st^ PC showed large temporal fluctuations (Figure 1B, Supplementary Figure S1A).

The large variance explained by the 1^st^ PC indicates that the spatiotemporal dynamics of brain activity in that window can be projected to a single mode, which indicates strong brain-wide synchronization (Vohryzek et al., 2020). We identified time windows showing temporally local maxima of the variance explained by the 1^st^ PC and named these windows as TSs (Figure 1B red dots). In TS, the 1^st^ PC of the TS dominated the temporal fluctuation of ROI timecourses, suggesting strong brain-wise synchronization in this window. To determine the best window size for detecting brain-wide synchronization, the timecourse of the 1^st^ PC was examined for each TS window (Supplementary Figure S1B). The timecourse monotonically increased or decreased with window sizes smaller than 25 TRs (corresponding to 18 s; Supplementary Figure S1B *left* and *middle*). With larger window sizes, the timecourses did not show a temporally consistent pattern or monotonic changes (Supplementary Figure S1B), suggesting that window sizes <25 TR were suitable for extracting brain-wide synchronization (Lindquist et al., 2014). Because the sign of PC cannot be determined uniquely, in order to align the timecourses of the 1^st^ PC across TSs, we flipped the signs of the PCs such that the PC timecourses showed a monotonic increase (Supplementary Figure S1C). Because the 1^st^ PC obtained in these procedures provided spatial patterns of globally synchronized resting-state brain activity, we denoted the 1^st^ PCs in TS as transient synchronization patterns (TSPs).

To characterize the timecourses of the variance explained by the 1^st^ PC (Figure 1B), we next examined these timecourses in relation to the quantitative measures for temporal characteristics used in previous studies. The timecourse of the variance explained by the 1^st^ PC was highly consistent with the global efficiency (Zalesky et al., 2014; Supplementary Figure S2 second top), Frobenius norm of the correlation matrix (Allen et al., 2014; Supplementary Figure S2 third top), and phase synchronization (Ponce-Alvarez et al., 2015; Senden et al., 2017; Supplementary Figure S2 bottom). However, the timecourse was not similar to the point process analysis (PPA) measure (Tagliazucchi et al., 2012; Liu and Duyn, 2013; Supplementary Figure S2 bottom). These results confirmed that sw-PCA captured the temporal characteristics of resting-brain activity.

### Classification of TSP

TSs were observed approximately 74.6 ± 4.2 times (mean ± SD) during one functional run (1,200 frames per run). To identify dominant patterns of activity dynamics during rest as observed in prior studies (Allen et al., 2014; Tagliazucchi and Laufs, 2014), we classified TSPs into 12 patterns by k-means clustering (Allen et al., 2014). TSPs corresponding to the centers of the patterns were extracted (Figure 1C).

We then examined the transition patterns of TSP among the 12 patterns (Supplementary Figure S3A). This revealed that the occurrence of a TSP was frequently followed by occurrence of another particular TSP. Thus, these two TSPs comprised a pair. Six pairs of patterns were identified, and each pair mutually transited over the TSP (Supplementary Figure S3A). Two TSPs belonging to the same cluster appeared almost equally (Supplementary Figure S3B). We labeled the center frame of TS as sub-clusters, and pairs of sub-clusters comprised a cluster (e.g., Cluster 1 is composed of Sub-cluster 1-1 and Sub-cluster 1-2). A total of 6 clusters and 12 sub-clusters were found (2 sub-clusters per cluster; Figure 1C). Very similar clusters were observed with different numbers of patterns used for the k-means clustering obtained (Supplementary Figures S3C, D), suggesting that the clusters were robust to the change of parameters in the analysis. Note that the present analysis did not reveal whether TSPs corresponded to states or attractors in the resting-brain activity; hence we intentionally avoided use of these terms (see Discussion).

### Reconstruction of voxel-based SAPs

To explore the detailed patterns of brain activations related to TS, we performed a variant of a standard event-related GLM analysis to reconstruct voxel-based maps associated with TSPs. The analysis coded TS as regressors, and voxel-wise parameter estimation was performed for each sub-cluster (Figure 1D; see also Methods). We named the reconstructed voxel-based maps representing brain-wide synchronization patterns as SAPs. Figure 2 shows the SAP for each sub-cluster. Note that SAPs of a pair of sub-clusters showed opposite spatial patterns, which was attributable to TSP signs.

Voxel-based representation of SAPs allowed us to perform decoding analysis on SAPs, which produced word clouds based on their topographical similarity to functional brain maps curated in previous meta-analyses (Figure 2; see also Methods; Yarkoni et al., 2011; Margulies et al., 2016; Chen et al., 2018; Gonzalez-Castillo et al., 2019; Matsui et al., 2022b). SAPs for Sub-clusters 1-1, 3-1, and 4-1 showed similarity to functional maps for motor movement and somatosensory stimulation. Because subjects were refrained from making movements during rs-fMRI in the HCP's protocol, the decoding results may reflect motor-intention or imagination rather than movements per se. SAPs for Sub-cluster 2-1 were characterized by vision-related terms such as “visual,”“eye,”“attentional,” “stimulus,” and “eye movements.” SAPs for Sub-cluster 5-1 were labeled as “working memory” and “executive.” For sub-clusters paired with those similar to functional maps of motor, visual, and executive functions [i.e., clusters labeled as “X-2,” (X: 1 to 6)], word clouds included “autobiographical,” “self-referential,” “mental states,” “theory minds,” and “cognitive control.” These higher mental functions are often associated with fronto-medial wall areas spanning the ventromedial prefrontal cortex to dorsal anterior cingulate cortex (Gusnard and Raichle, 2001; Kelley et al., 2002; Gallagher and Frith, 2003; Piefke et al., 2003; Rushworth et al., 2004; Mitchell et al., 2005; Summerfield et al., 2009).

### Relationships between cluster occurrence and RSFC

We further examined the relationships between (static) RSFC and TSP. If TSs involving two RSNs (e.g., motor/sensory and visual, like Cluster 1-1) in its TSP occurred in a scanning run, high temporal correlation between the two RSNs should be found. To confirm this possibility, we performed multiple regression analysis where the strengths of RSFC (13 × 12/2 = 78 combinations) were predicted by the co-occurrence of each cluster, and R-squared values of the regression were calculated. The cluster occurrence explained well the strength of static RSFC among many of the networks including sensory, visual, cingulo-opercular, auditory, ventral attention, and cerebellar networks (Supplementary Figure S4A).

The strength of RSFC was also predicted by SAP. SAP explained well the FC strength among those networks (Supplementary Figure S4B). When the regression analysis was based on the SAP of each cluster separately, Clusters 4 and 6 were found to better explain the strengths of RSFC (Supplementary Figure S4C). However, FPN and DMN showed smaller explained variances [red arrowheads (a/b) in Supplementary Figure S4B] [DMN (a): r^2^ = 0.3; FPM (b): r^2^ = 0.2]. These results confirmed that cluster occurrence and SAPs could explain the characteristics of static RSFC.

### Examination of clusters

The clusters extracted above may be a statistical artifact due to sampling variability rather than reflecting distinct brain states (Laumann et al., 2017: Liegeois et al., 2017; Matsui et al., 2022a). Therefore, we tested whether similar clusters were found in null data, which was constructed to be stationary (Liegeois et al., 2017). We used phase randomized data as null data, which kept the auto-/cross-covariance of the real data (Calhoun et al., 2014). TSPs calculated based on the null data were quite similar to those calculated based on the original data. Spatial correlation between STPs of real and null data was high (Supplementary Figure S5A), similar to prior studies (Laumann et al., 2017; Matsui et al., 2022a).

However, internal cluster validity index (Calhoun et al., 2014) differed between null and real data (Supplementary Figure S5B), suggesting that the cluster structure of TSPs was not fully explained by lag cross correlation of fMRI signals and that these clusters may correspond to distinct states constituting non-stationary resting-brain activity (Calhoun et al., 2014).

### Temporal characteristics of cluster occurrences

**Figure 3:**
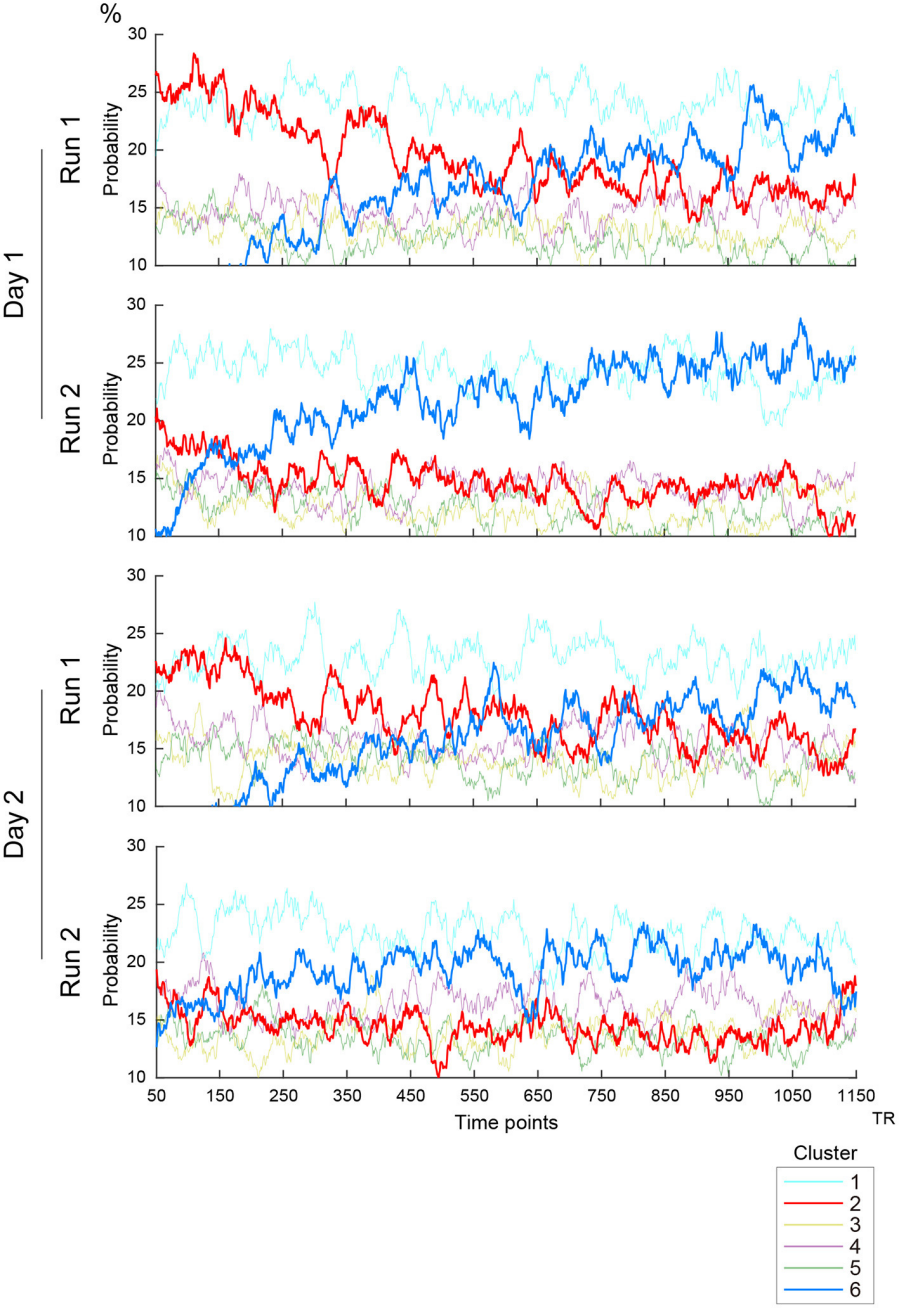
Timecourses of the occurrence of clusters. Vertical and horizontal lines indicate occurrence probability and time, respectively. Line colors indicate clusters as labeled in the right inset. Four scanning runs were administered in two days, and the panels show the timecourses of the first and second scanning runs on Days 1 and 2.

To examine further whether the occurrences and transition properties of the clusters changed during a scanning run, we calculated ensemble mean probabilities across subjects for each RS scanning run (2 runs × 2 days; Figure 3). A gradual increase of Cluster 6 occurrences and high occurrence of Cluster 2 in the first 200 frames (approximately 2–3 min) were observed on both scanning days. These observations are consistent with prior studies (Allen et al., 2014; Abrol et al., 2016, 2017). However, Cluster 6 occurrences increased in earlier frames in the second run than in the first run on both Days 1 and 2. Additionally, high occurrences of Cluster 2 were observed in the first 200 frames particularly in the first run on each of Days 1 and 2. Importantly, these results were not observed for cluster occurrences calculated from the null data (Supplementary Figure S5C).

**Figure 4:**
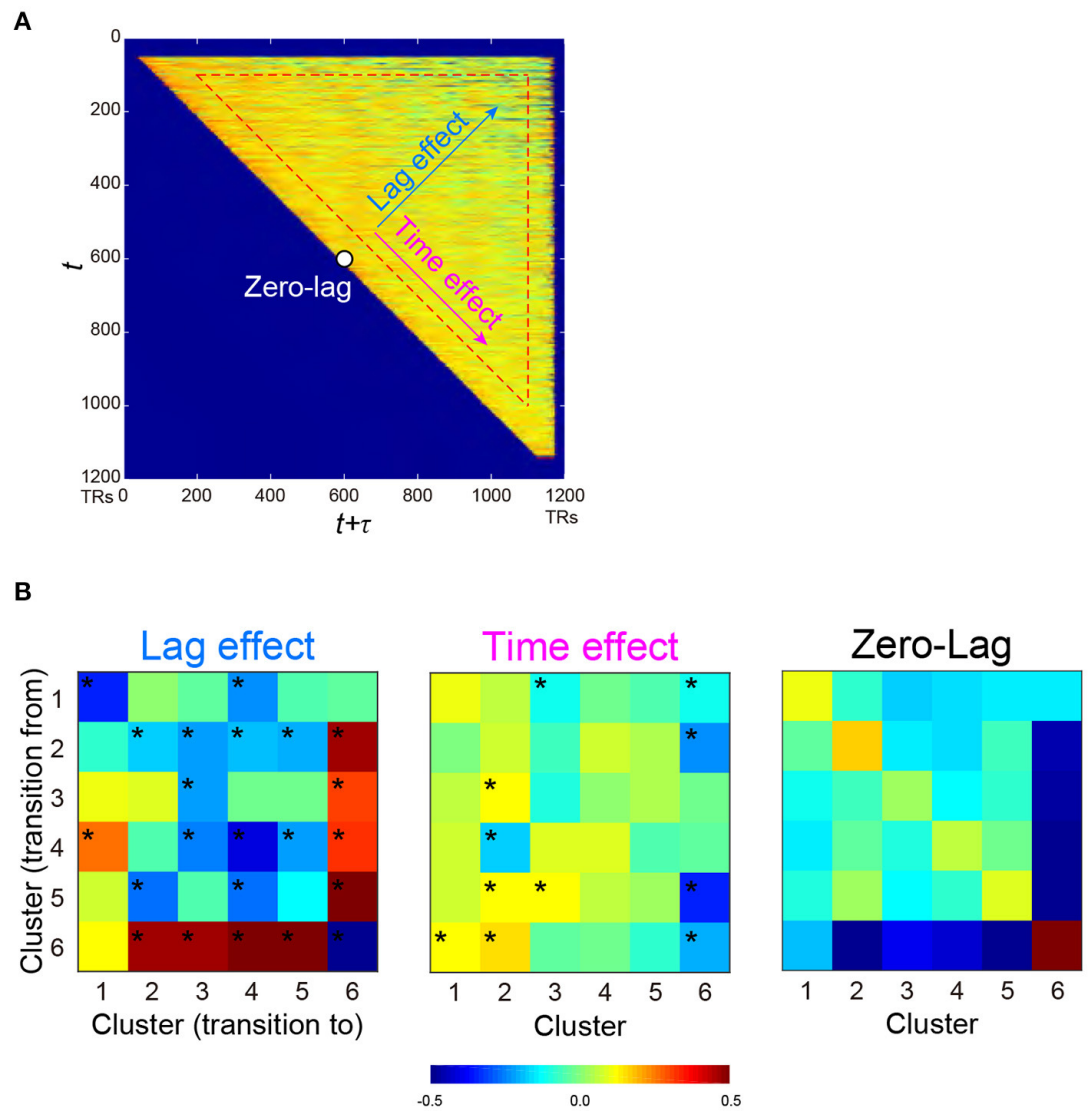
PMI of occurrence probability reflects the temporal relationships of occurrences between two clusters. PMI was calculated at time *t* and *t* + τ, which provided a PMI matrix **(A)**. A PMI matrix was created for each combination of the 6 clusters, and each matrix was decomposed into lag (τ) effect, time (*t*) effect, and zero-lag (constant) effect based on multiple regression analysis. **(B)** Regression coefficients for all cluster combinations are color-coded for lag effect (*left*), time effect (*middle*), and zero-lag (*right*), as shown at the bottom. ^*^P < 0.05, FWE-corrected for multiple comparison.

Next, we examined the temporal structure of cluster occurrence probability by calculating temporal point-wise mutual information (PMI; Church and Hanks, 1989). In the current study, PMI indicated the probability of observing a cluster at time *t* + τ given a cluster occurring at time *t* (See also Methods). Then, for all combinations of clusters, PMI was calculated for all *t* and τ (*t* > 0; τ > 0; *t* + τ <1,200), providing a PMI matrix (Figure 4A). To examine the temporal structure of the PMI matrix, it was fitted by a multiple linear regression model involving three explanatory variables, namely, *t* (time effect), τ (lag effect) and a constant (zero-lag PMI) (Figure 4A). The parameters were then estimated for all cluster combinations (Figure 4B). Statistical significance was tested based on phase randomized null data (Supplementary Figures S5C, D; Hindriks et al., 2016; Laumann et al., 2017). Note that rejection of the null hypothesis (FWE-corrected *P* < 0.05) suggests that these temporal structures of cluster occurrences cannot be fully explained by autocorrelations of rs-fMRI signals.

Lag effects showed large negative effects in diagonal components (Figure 4B *left*). Clusters 2 and 6 showed strong negative lag effects, indicating that Clusters 6 and 2 alternated in short time intervals. However, Cluster 6 showed greater positive lag effects with other Clusters 1-5, indicating that Cluster 6 and other clusters infrequently occurred especially in a short time interval. Note that small tau effects (τ <100 TRs) and early (first 100 frames) and late (last 100 frames) scanning periods were eliminated from fitting (see Methods). This was because the current analysis focused on cluster co-occurrences in long periods, and cluster co-occurrences in short periods were derived from autocorrelation of temporally filtered rs-fMRI signals.

It is still possible that these PMI changes were confounded by the change in cluster occurrence probability during early and late periods of a scanning run. For example, a positive lag effect between Clusters 2 and 6 (Figure 4B *left*) was derived from the frequent occurrence of Cluster 2 and 6 in the early and later periods, respectively (Figure 3). This time effect of PMI was unlikely to be attributable to the limited sample size in those scanning periods, because the time effects of PMI from other clusters to Cluster 6 were low (Figure 4B *middle*). To confirm this effect further, we performed a simulation analysis in which randomized null data sets preserved the temporal characteristics of cluster occurrence frequency of the real data (Supplementary Figures S6A–C). This control analysis confirmed that these lag and time effects of PMI in the real data were not affected by the temporal changes of cluster occurrence (Supplementary Figure S6D).

### Relationships between clusters and task-related functional maps

**Figure 5:**
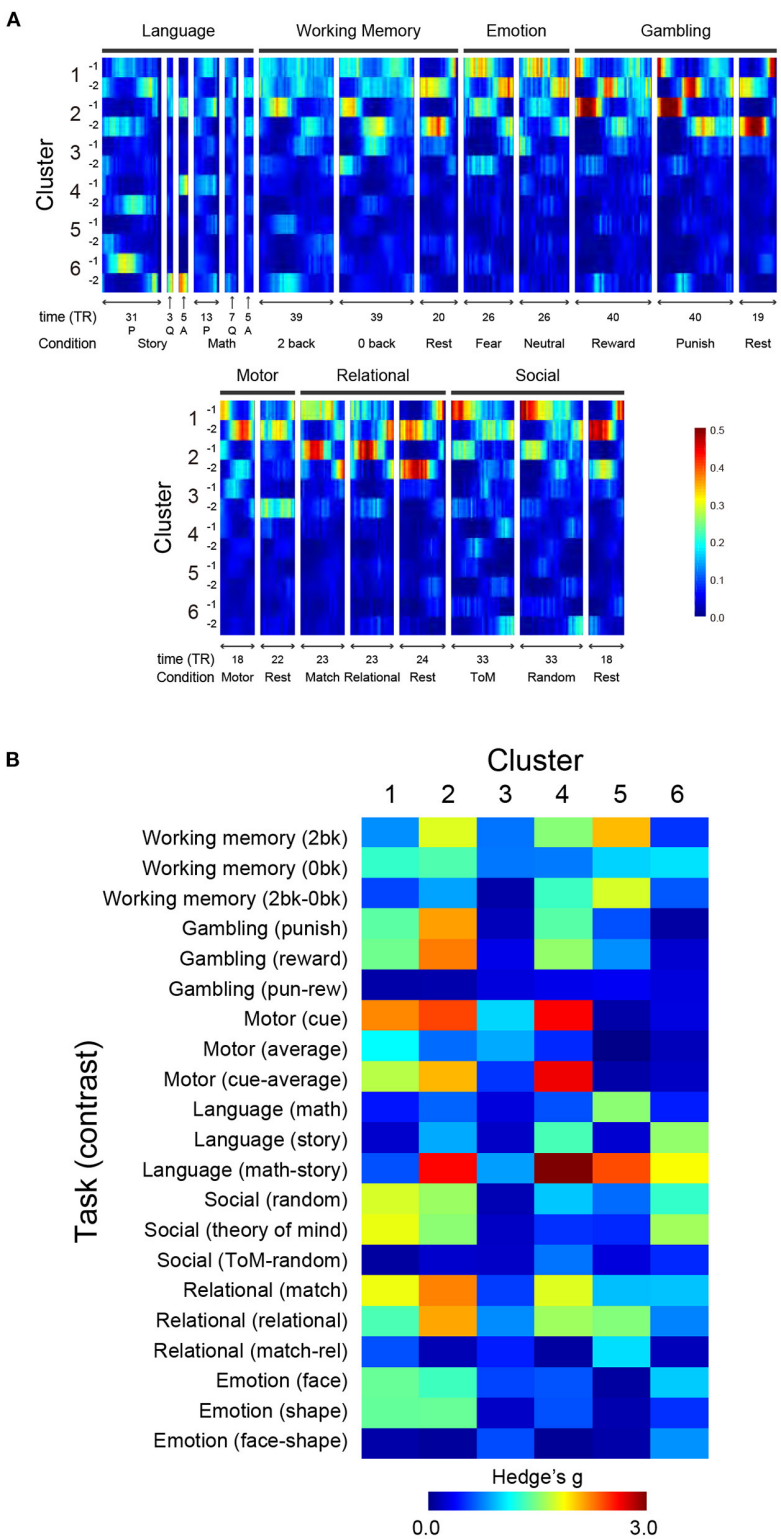
Relationships of TSP in resting state and task states. **(A)** TSPs during performance of the 7 tasks were classified using a classifier trained on resting-state data, and classification scores of the task-TSPs were calculated along task blocks. For each cluster and scanning frame, scores are color-coded as shown at the bottom right. For language tasks, P and Q denote presentation onset of problems and questions for story/math trials, respectively, and A denotes response onsets. **(B)** Spatial consistency of TSPs between during resting state and task performance. Spatial consistency was calculated as correlation indexed by Hedges' g between TSP maps. The effect size of Cluster N-1 and N-2 was averaged and then color-coded on the heat map.

To examine if clusters identified in the resting state also appear in the task state, we compared TSPs during resting state (Figure 1C) and those during task states. We first calculated TSPs during task-fMRI scans (task-TSPs) available from HCP (7 behavioral tasks; Barch et al., 2013). Task-TSPs were extracted using the same procedure used to extract TSPs in resting state. Then, task-TSPs were classified by the k-nearest neighbor method (k = 5), in which the classifier was trained based on TSPs in resting state. Large classification scores indicate that task-TSPs are spatially similar to the TSPs in resting state. Figure 5A shows timecourses of classification scores of 7 task-TSPs along task blocks for each cluster defined by resting-state TSPs. Classification scores of Clusters 1 (1-1 and 1-2) and 2 (2-1 and 2-2) were consistently high across the 7 tasks. However, the score of Cluster 1 was higher during motor and social recognition task blocks than for working memory and relational tasks. Cluster 2 showed the opposite pattern, possibly suggesting that Cluster 2 is related to cognitive demands.

Next, we compared the spatial patterns of task-TSPs and TSPs in resting state. To allow voxel-level comparison, we examined spatial correlations between SAPs (Figure 2) and task activation maps (see Methods). Statistical significance was tested by estimating a null distribution of the correlations based on the permutation of subjects. Cumulative distributions of correlations for each SAP and task-related activation map were statistically evaluated using the null distribution (Supplementary Figure S7A).

Figure 5B shows effect size (Hedge's d) of the spatial correlation. The maps were largely consistent for Clusters 1 and 2, which showed strong positive or negative correlations with major task events. Specifically, Cluster 2 showed strong correlations with working memory events, gambling events, and a language contrast for math vs. story. Clusters 4, 5, and 6 showed strong correlations with various tasks and contrasts; Cluster 4 with motor cue events and a language task contrast (math vs. story); Cluster 5 with a working memory contrast (2-back vs. 0-back), a language contrast (math vs. story), and relational events; and Cluster 6 with language and social tasks. The direction of the correlation was reversed between Sub-clusters (e.g., X-1 and X-2) (Supplementary Figure S7B). Thus, voxel-based reconstruction of SAPs allowed a detailed comparison with task-evoked activity, which is useful for interpreting the clusters found in the resting state.

### Relationships between clusters and behavioral characteristics

**Figure 6:**
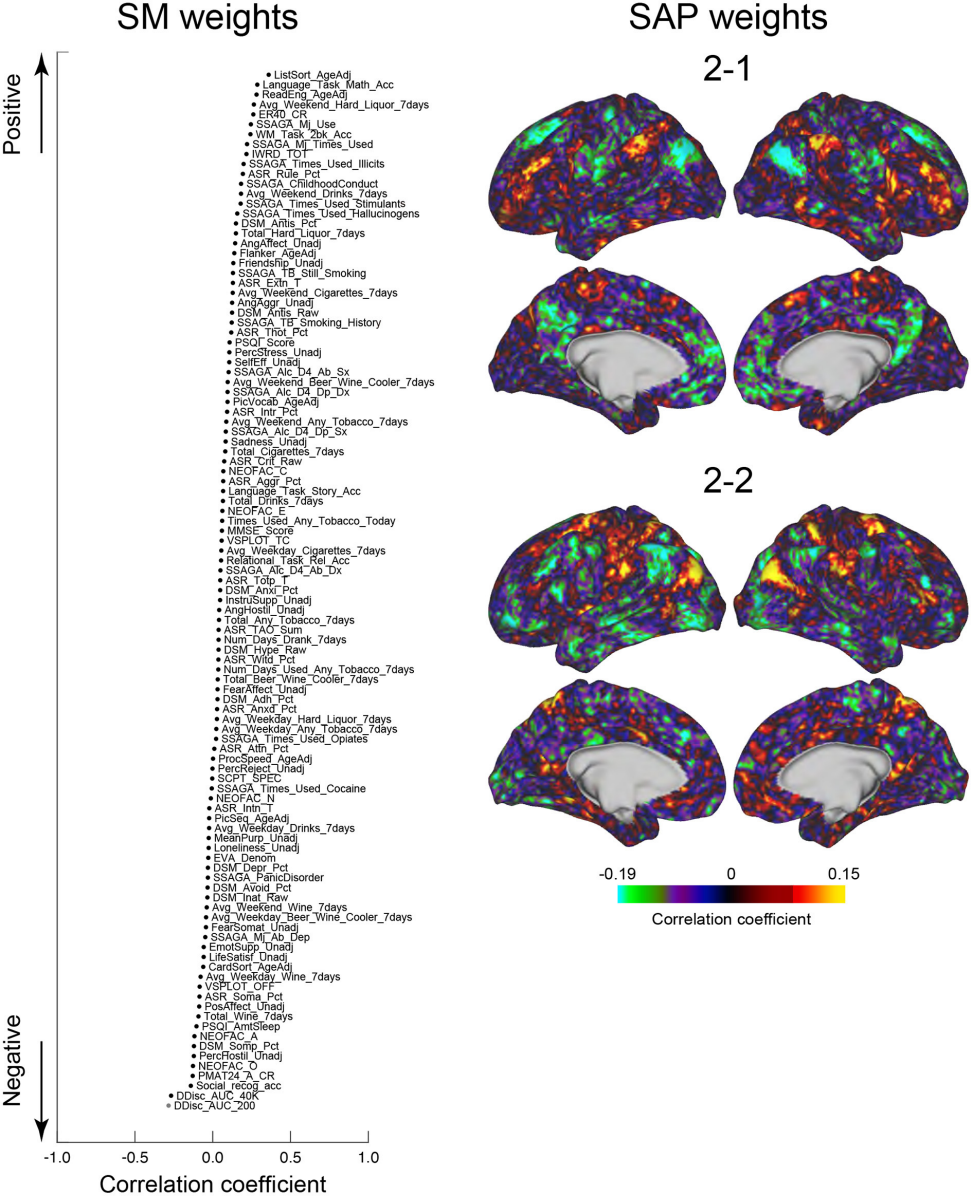
CCA analysis of SMs and Cluster 2. SM weights of CCA-mode **(left)**. The horizontal axis indicates the correlation coefficient to CCA-mode. The vertical axis indicates SMs for positive (top) and negative (bottom) directions. A whole-brain map is shown by TSP weight **(right)**. The color indicates the correlation coefficient with CCA-mode.

**Figure 7:**
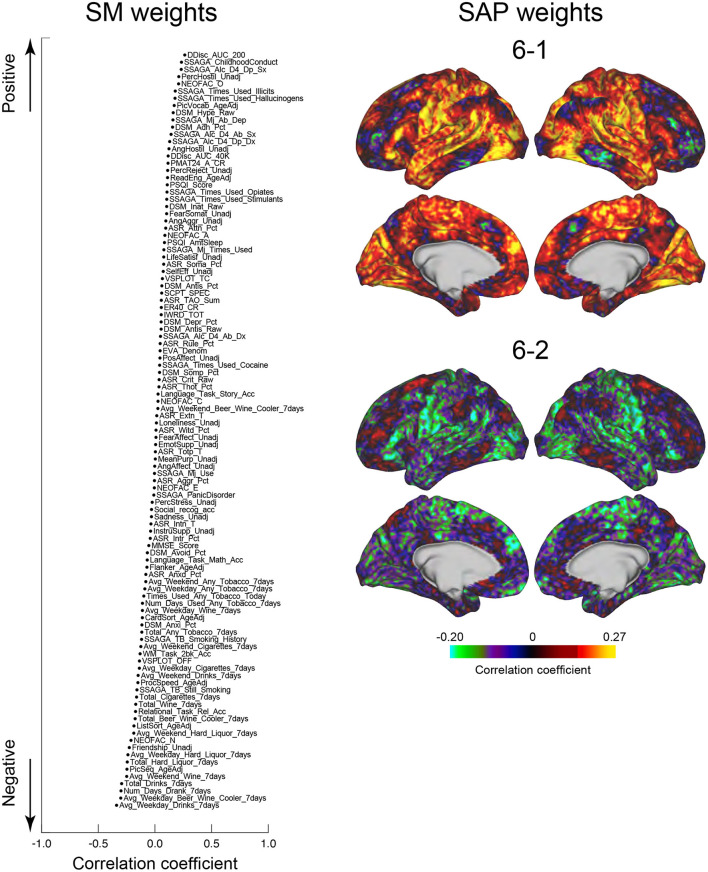
CCA analysis of SMs and Cluster 6. Formats are similar to those in Figure 6.

We next tested whether the clusters identified in the current study were associated with SMs. Similar to the previous studies (Smith et al., 2015; Miller et al., 2016; Bijsterbosch et al., 2017), we examined correlations between SMs and SAPs using CCA (Hotelling, 1936). Clusters 2 and 6 showed significant correlation with CCA mode (Ps: *P* < 0.05; FWE corrected; Figures 6, 7). For Cluster 2, SM weights were positively correlated with working memory, language, drinking, and drug use but negatively correlated with self-regulation (control) and fluid intelligence (Figure 6 left). The weight map of SAP (Figure 6 right) showed a spatial pattern similar to SAPs for Sub-clusters 2-1 and 2-2 (Figure 2), suggesting that subjects with greater CCA mode show a more prominent pattern for Cluster 2. For Cluster 6, scores for self-control, drug use, CCD, and alcohol dependence or abuse showed positive weights, but negative weights were observed for frequency of drinking (Figure 7 left). Weight maps for SAP (Figure 7 right) showed a spatial pattern similar to those of SAPs for Sub-clusters 6-1 and 6-2 (Figure 2), suggesting that individuals with greater CCA showed a prominent pattern for Cluster 6.

## Discussion

The current study examined the transient spatiotemporal structure of rs-fMRI data and identified temporal synchronization patterns consisting of a set of clusters occurring periodically within the range of seconds to minutes. Activation patterns associated with the clusters showed spatial patterns similar to those observed during task performance. These activation patterns and their occurrence were also associated with individuals' behavioral characteristics.

The analysis procedure developed in the current study would be useful to examine the spatiotemporal characteristics of rs-fMRI data of patients with psychiatric and neurological disorders (Buckner et al., 2008; Du et al., 2016). The present method allows comparison between RSNs and time-resolved dynamics in rs-fMRI data at a voxel-level resolution. Nevertheless, the current study had some limitations. Our analysis was based on a sliding window approach, and the window size was set to 25 TRs (18 s) somewhat arbitrarily. We also used a predetermined number of patterns to extract TSPs using the k-means algorithm. The current results demonstrated that our approach was powerful to examine the spatiotemporal characteristics of resting-state fMRI signal. However, we acknowledge that other approaches could be effective (Abrol et al., 2017; Bassett and Sporns, 2017; Laumann et al., 2017).

Prior studies examining the spatiotemporal characteristics of rs-fMRI signals provided a helpful interpretation of time-resolved states (Allen et al., 2014; Calhoun et al., 2014; Abrol et al., 2017; Nomi et al., 2017; Vergara et al., 2017; Vidaurre et al., 2017). The current study extended this understanding by providing a new method to annotate the time-resolved activity patterns based on their relationships to task-evoked activation patterns and behavioral characteristics by using voxel-based maps (i.e., SAPs). The correlations between SAPs and SMs suggest that the time-resolved network structure is reflected in the behavior and psychological characteristics of individual subjects. Further exploration and examination are possible based on supervised classification to decode a participant's mental state from static RSFC (Solovey et al., 2015; Kragel et al., 2016; Kucyi et al., 2017; Fortenbaugh et al., 2018).

Our proposed method differs from other existing methods for the analysis of spatiotemporal structures of resting-state fMRI. Whereas previous methods such as temporal functional modes (TFM) (Smith et al., 2012) and lag threads (Mitra et al., 2015) extract independent or orthogonal modules of spatiotemporal activities, the present method focus on moments of low-dimensional dynamics as quantified by sw-PCA. The present method also differs from the cross-hierarchy propagating waves (Gu et al., 2021) which specifically extracted activity propagation along the principal gradient of the cerebral cortex (Margulies et al., 2016). Unlike the cross-hierarchy propagating waves, the proposed method in principle allows detection of standing waves. The quasi-periodic pattern (QPP) extracts spatiotemporal activity patterns using a template-matching approach (Majeed et al., 2011). In contrast to QPP that uses sliding-window analysis to iteratively extract single spatiotemporal activity pattern, the present method allows simultaneous detection of multiple activity patterns. The present method shares essential idea with the Leading Eigenvector Dynamic Analysis (Vohryzek et al., 2020) that extract whole-brain BOLD phase-locking patterns from ROI-based fMRI data. In the present method, we additionally devised a GLM-based method that can visualize the event-related activity patterns at a voxel level. This voxel-based visualization allowed us to interpret the activity patterns using functional decoders. Because we aimed to extract transient brain activity time-locked to TSP events and then explored brain regions showing the transient activity, we used a standard GLM approach assuming that the event-locked transient activity shows canonical hemodynamic impulse response after an event, rather than an assumption-free method such as a finite impulse response modeling.

### Characterization of clusters

The present method allowed interpretation of the clusters based on their spatiotemporal characteristics, their relationships with task-evoked activations, and correlation with SMs (Supplementary Table S1). These results naturally lead to the interpretation that these clusters correspond to brain states that temporally change during rs-fMRI scanning (Leonardi et al., 2013; Allen et al., 2014). However, such temporal characteristics may also reflect sampling variability (Laumann et al., 2017), evoked attention, head motion (Power et al., 2017) and/or sleep (Tagliazucchi and Laufs, 2014; Stoffers et al., 2015; Hindriks et al., 2016; Laumann et al., 2017). Recent studies showed that not only the spatial patterns but also temporal dynamics of the putative brain states were reproducible in null data that were stationary by construction (Liegeois et al., 2017; Matsui et al., 2022a). Although some temporal aspects of the clusters in the present study were not fully explained by null data (Supplementary Figure S5B), as in some previous studies (Allen et al., 2014; Matsui et al., 2019), the differences were small. Therefore, in the present study, we avoided the interpretation that the clusters represented brain states.

Nevertheless, it is still important and useful to consider the relationship between clusters and functional networks involved in task performance. Cluster 1 may reflect general task processing (Dosenbach et al., 2006, 2008), because TSPs similar to Cluster 1 were observed in a wide range of tasks during fMRI scanning (Figure 5). Cluster 6 may reflect drowsiness, because the occurrence of this cluster was related to sleep score (Figure 5). The spatial pattern of Cluster 6 also implied subcortical-cortical anti-correlation (Figure 2). A gradual increase of occurrence of Cluster 6 was also observed during scanning (Figure 3), consistent with a prior report [State 3 in Allen et al. (2014)].

All these interpretations involve some degree of speculation because the results were based on association (not causal) analyses between spatiotemporal characteristics during resting state and those during task states or individual characteristics. Classification of clusters (6 in the current study) also involves a certain degree of arbitrariness. Therefore, more precise mental events or states would be identified if the clusters were classified and examined in more detail. Compared to ROI-based approaches (Vohryzek et al., 2020), the present voxel-level approach would be advantageous for obtaining such detailed characterizations.

The clusters comprised of pairs of sub-clusters are labeled differentially by functional terms even though the sub-clusters are occurring in close temporal proximity. The appearance of the paired sub-clusters is temporally close because monotonic signal increase time-locked to a TSP event is followed by a monotonic decrease in which another TSP event can occur. Most likely because this pattern arises from the slow nature of signal fluctuations, the TSP events of paired sub-clusters show inverse activity patterns (Figure 2). This entails that the paired sub-clusters involve mutually exclusive brain regions; therefore, they are labeled by differential functional terms.

It should be noted that, despite extensive denoising (e.g., FIX cleaning), BOLD signals used in the present study likely contain physiological (non-neuronal) noise. Such physiological noise may have affected the event-based analysis. Nevertheless, recent studies using simultaneous optical recording of neuronal and hemodynamic signals in mice reported that many transient events of neuronal activity were indeed visible in hemodynamics (Ma et al., 2016; Matsui et al., 2016, 2018). These studies support the interpretation that the events detected in the present study reflect underlying neuronal activity.

### Temporal patterns of cluster occurrence and static RSFC

As stated above, we found a close correspondence between the clusters in the resting state and cognitive process, attention, drowsiness and/or head motion. Interestingly, these clusters dominated more than half of TSs in the resting state (Supplementary Figure S3B). This result is consistent with a recent study demonstrating that behavioral events dramatically changed RSFC of the mouse brain (Winder et al., 2017). Nonetheless, it is natural to assume that psychological conditions and/or mental states of subjects during resting-state scanning affect static RSFC (Fox et al., 2005; Power et al., 2011). Indeed, we found that static RSFC was modulated by the temporal occurrence and dominance of the clusters (Supplementary Figure S4).

In contrast, DMN or FPN identified by static RSFC (Fox et al., 2005; Power et al., 2011) were stable and not affected by cluster occurrence. The temporal stability of DMN and FPN against TSP was consistent with the clustering results showing that the two networks were retained within single clusters (Figures 1C, 2). Therefore, the stable RSFC of DMN and FPN may reflect strong structural connectivity (Gusnard and Raichle, 2001). Alternatively, it may reflect task-related generic activation occurring during resting-state scans (Dosenbach et al., 2006; Cole et al., 2016; Shine et al., 2016a,b).

There is a possibility that the choice of a particular window size limited the analysis of FC transitions within a limited dynamic range. For example, FC transitions occurring at a shorter time scale (<25 TR) or at a much longer time scale (>25 TR) maybe undetected by the current choice of the window size. Nevertheless, our comparison of different window size (Supplementary Figure S1B) suggests this is unlikely to be the case. Supplementary Figure S1B shows time course of TSP (1^st^ PC) within a window, for various window sizes. For window size smaller than 25 TR, TSP time course showed monotonic change within a window, suggesting that resting-brain activity did not show sharp transitions at fast (<25 TR) time scale. In contrast, for window size larger than 35 TR, TSP time course did not show monotonic change, suggesting that the resting-brain activity showed activity change at a time scale shorter than these window sizes. Taken together, while we acknowledge a methodological limitation in sensitive dynamic range, the present data suggest that the current choice of window size was appropriate for capturing dominant FC transitions.

### Classification of behavior by *post-hoc* clustering

We observed repeated occurrences of TSs, which may reflect physiological functions. The current clustering and voxel-based reconstruction approach are useful techniques to understand the physiological processes underlying rs-fMRI. Indeed, the current study demonstrated that spatiotemporal patterns during TS were related to those during task engagement and the behavioral/psychological characteristics of individual subjects. These results suggest a potential link between individual moments during rs-fMRI and functional processes taking place in that moment. Because it is almost impossible to keep subjects' mental state stable during rs-fMRI, our approach to cluster TS events determined in a *post-hoc* manner provides a helpful way to examine the nature of the resting state.

## Data availability statement

The data set analyzed in the study is publicly available from Human Connectome Projects (https://www.humanconnectome.org/). Further inquiries can be directed to the corresponding authors.

## Ethics statement

Ethical review and approval was not required for the study on human participants in accordance with the local legislation and institutional requirements. Written informed consent from the patients/participants or patients/participants' legal guardian/next of kin was not required to participate in this study in accordance with the national legislation and the institutional requirements.

## Author contributions

YN designed the study and performed data analyses. YN, RL, TM, and KJ wrote the manuscript. All authors contributed to the article and approved the submitted version.

## References

[B1] Abrol A. Chaze C. Damaraju E. Calhoun V. D. (2016). “The Chronnectome: Evaluating replicability of dynamic connectivity patterns in 7500 resting fMRI datasets,” in 2016 38th Annual International Conference of the IEEE Engineering in Medicine and Biology Society (EMBC) (IEEE) 5571–5574. 10.1109/EMBC.2016.7591989 28269517

[B2] Abrol A. Damaraju E. Miller R. L. Stephen J. M. Claus E. D. Mayer A. R.. (2017). Replicability of time-varying connectivity patterns in large resting state fMRI samples. NeuroImage 163, 160–176. 10.1016/j.neuroimage.2017.09.020 28916181 PMC5775892

[B3] Allen E. A. Damaraju E. Plis S. M. Erhardt E. B. Eichele T. Calhoun V. D. (2014). Tracking whole-brain connectivity dynamics in the resting state. Cerebl. Cortex 24, 663–676. 10.1093/cercor/bhs352 23146964 PMC3920766

[B4] Barch D. M. Burgess G. C. Harms M. P. Petersen S. E. Schlaggar B. L. Corbetta M.. (2013). Function in the human connectome: Task-fMRI and individual differences in behavior. NeuroImage 80, 169–189. 10.1016/j.neuroimage.2013.05.033 23684877 PMC4011498

[B5] Bassett D. S. Sporns O. (2017). Network neuroscience. Nat. Neurosci. 20, 353–364. 10.1038/nn.4502 28230844 PMC5485642

[B6] Betzel R. F. Fukushima M. He Y. Zuo X. N. Sporns O. (2016). Dynamic fluctuations coincide with periods of high and low modularity in resting-state functional brain networks. NeuroImage 127, 287–297. 10.1016/j.neuroimage.2015.12.001 26687667 PMC4755785

[B7] Bijsterbosch J. Harrison S. Duff E. Alfaro-Almagro F. Woolrich M. Smith S. (2017). Investigations into within- and between-subject resting-state amplitude variations. Neuroimage 159, 57–69. 10.1016/j.neuroimage.2017.07.014 28712995 PMC5678294

[B8] Biswal B. Yetkin F. Z. Haughton V. M. Hyde J. S. (1995). Functional connectivity in the motor cortex of resting human brain using echo-planer MRI. Magn. Res. Med. 34, 537–541. 10.1002/mrm.1910340409 8524021

[B9] Buckner R. L. Andrews-Hanna J. R. Schacter D. L. (2008). The brain's default network - Anatomy, function, and relevance to disease. Ann. N. Y. Acad. Sci. 1124, 1–38. 10.1196/annals.1440.011 18400922

[B10] Calhoun V. D. Miller R. Pearlson G. Adali T. (2014). The chronnectome: time-varying connectivity networks as the next Frontier in fMRI data discovery. Neuron 84, 262–274. 10.1016/j.neuron.2014.10.015 25374354 PMC4372723

[B11] Chang C. Glover G. H. (2010). Time-frequency dynamics of resting-state brain connectivity measured with fMRI. NeuroImage 50, 81–98. 10.1016/j.neuroimage.2009.12.011 20006716 PMC2827259

[B12] Chen R. H. Ito T. Kulkarni K. R. Cole M. W. (2018). The human brain traverses a common activation-pattern state space across task and rest. Brain Connect. 8, 429–443. 10.1089/brain.2018.0586 29999413 PMC6152856

[B13] Church K. W. Hanks P. (1989). Word association norms, mutual information, and lexicography. Comput. Ling. 16, 22-29. 10.3115/981623.981633

[B14] Cole M. W. Ito T. Bassett D. Schultz D. H. (2016). Activity flow over resting-state networks shapes cognitive task activations. *Nat*. Neurosci. 12, 1718–1726. 10.1038/nn.4406 27723746 PMC5127712

[B15] Damaraju E. Allen E. A. Belger A. Ford J. M. McEwen S. Mathalon D. H.. (2014). Dynamic functional connectivity analysis reveals transient states of dysconnectivity in schizophrenia. NeuroImage Clin. 5, 298–308. 10.1016/j.nicl.2014.07.003 25161896 PMC4141977

[B16] Damoiseaux J. S. Rombouts S. Barkhof F. Scheltens P. Stam C. J. Smith S. M.. (2006). Consistent resting-state networks across healthy subjects. *Proc. Natl. Acad. Sci*. USA. 103, 13848–13853. 10.1073/pnas.0601417103 16945915 PMC1564249

[B17] Dosenbach N. U. F. Fair D. A. Cohen A. L. Schlaggar B. L. Petersen S. E. (2008). A dual-networks architecture of top-down control. Trends Cogn. Sci. 12, 99–105. 10.1016/j.tics.2008.01.001 18262825 PMC3632449

[B18] Dosenbach N. U. F. Visscher K. M. Palmer E. D. Miezin F. M. Wenger K. K. Kang H. S. C.. (2006). A core system for the implementation of task sets. Neuron 50, 799–812. 10.1016/j.neuron.2006.04.031 16731517 PMC3621133

[B19] Du Y. H. Pearlson G. D. Yu Q. B. He H. Lin D. D. Sui J.. (2016). Interaction among subsystems within default mode network diminished in schizophrenia patients: A dynamic connectivity approach. Schiz. Res. 170, 55–65. 10.1016/j.schres.2015.11.021 26654933 PMC4707124

[B20] Faghiri A. Stephen J. M. Wang Y. P. Wilson T. W. Calhoun V. D. (2018). Changing brain connectivity dynamics: From early childhood to adulthood. Hum. Brain Mapp. 39,1108–1117. 10.1002/hbm.23896 29205692 PMC5807176

[B21] Fortenbaugh F. C. Rothlein D. McGlinchey R. DeGutis J. Esterman M. (2018). Tracking behavioral and neural fluctuations during sustained attention: A robust replication and extension. Neuroimage 171, 148–164. 10.1016/j.neuroimage.2018.01.002 29307606 PMC5857436

[B22] Fox M. D. Qian T. Y. Madsen J. R. Wang D. H. Li M. L. Ge M. L.. (2016). Combining task-evoked and spontaneous activity to improve pre-operative brain mapping with fMRI. NeuroImage 124, 714–723. 10.1016/j.neuroimage.2015.09.030 26408860 PMC4831649

[B23] Fox M. D. Raichle M. E. (2007). Spontaneous fluctuations in brain activity observed with functional magnetic resonance imaging. Nat. Rev. Neurosci. 8, 700–711. 10.1038/nrn2201 17704812

[B24] Fox M. D. Snyder A. Z. Vincent J. L. Corbetta M. Van Essen D. C. Raichle M. E. (2005). The human brain is intrinsically organized into dynamic, anticorrelated functional networks. Proc. Natl. Acad. Sci. USA. 102, 9673–9678. 10.1073/pnas.0504136102 15976020 PMC1157105

[B25] Gallagher H. L. Frith C. D. (2003). Functional imaging of ‘theory of mind'. Trends Cogn. Sci. 7, 77–83. 10.1016/S1364-6613(02)00025-6 12584026

[B26] Glasser M. F. Smith S. M. Marcus D. S. Andersson J. L. Auerbach E. J. Behrens T. E.. (2016). The human connectome project's neuroimaging approach. Nat. Neurosci. 19, 1175–1178. 10.1038/nn.4361 27571196 PMC6172654

[B27] Glasser M. F. Sotiropoulos S. N. Wilson J. A. Coalson T. S. Fischl B. Andersson J. L.. (2013). The minimal preprocessing pipelines for the Human Connectome Project. Neuroimage 80, 105–124. 10.1016/j.neuroimage.2013.04.127 23668970 PMC3720813

[B28] Gonzalez-Castillo J. Caballero-Gaudes C. Topolski N. Handwerker D. A. Pereira F. Bandettini P. A. (2019). Imaging the spontaneous flow of thought: Distinct periods of cognition contribute to dynamic functional connectivity during rest. Neuroimage 202, 116129. 10.1016/j.neuroimage.2019.116129 31461679 PMC6819261

[B29] Griffanti L. Salimi-Khorshidi G. Beckmann C. F. Auerbach E. J. Douaud G. Sexton C. E.. (2014). ICA-based artefact removal and accelerated fMRI acquisition for improved resting state network imaging. NeuroImage 95, 232–247. 10.1016/j.neuroimage.2014.03.034 24657355 PMC4154346

[B30] Gu Y. Sainburg L. E. Juang S. Han F. Williams J. W. Kiu Y.. (2021). Brain activity fluctuations propagate as waves traversing the cortical hierarchy. *Cereb*. Cortex 31, 3986–4005. 10.1093/cercor/bhab064 33822908 PMC8485153

[B31] Gusnard D. A. Raichle M. E. (2001). Searching for a baseline: Functional imaging and the resting human brain. Nat. Rev. Neurosci. 2, 685–694. 10.1038/35094500 11584306

[B32] Handwerker D. A. Roopchansingh V. Gonzalez-Castillo J. Bandettini P. A. (2012). Periodic changes in fMRI connectivity. NeuroImage 63, 1712–1719. 10.1016/j.neuroimage.2012.06.078 22796990 PMC4180175

[B33] Hansen E. C. A. Battaglia D. Spiegler A. Deco G. Jirsa V. K. (2015). Functional connectivity dynamics: Modeling the switching behavior of the resting state. NeuroImage 105, 525–535. 10.1016/j.neuroimage.2014.11.001 25462790

[B34] Hentschke H. Stüttgen M. C. (2011). Computation of measures of effect size for neuroscience data sets. Eur. J. Neurosci. 34, 1887–1894. 10.1111/j.1460-9568.2011.07902.x 22082031

[B35] Hindriks R. Adhikari M. H. Murayama Y. Ganzetti M. Mantini D. Logothetis N. K.. (2016). Can sliding-window correlations reveal dynamic functional connectivity in resting-state fMRI? NeuroImage 127, 242–256. 10.1016/j.neuroimage.2015.11.055 26631813 PMC4758830

[B36] Hotelling H. (1936). Relations between two sets of variates. Biometrika 28, 321–377. 10.1093/biomet/28.3-4.321

[B37] Hutchison R. M. Womelsdorf T. Allen E. A. Bandettini P. A. Calhoun V. D. Corbetta M.. (2013). Dynamic functional connectivity: Promise, issues, and interpretations. NeuroImage 80, 360–378. 10.1016/j.neuroimage.2013.05.079 23707587 PMC3807588

[B38] Jones D. T. Vemuri P. Murphy M. C. Gunter J. L. Senjem M. L. Machulda M. M.. (2012). Non-stationarity in the “resting brain's” modular architecture. PLoS ONE 7, e39731. 10.1371/journal.pone.0039731 22761880 PMC3386248

[B39] Keerativittayayut R. Aoki R. Sarabi M. T. Jimura K. Nakahara K. (2018). Large-scale network integration in the human brain tracks temporal fluctuations in memory encoding performance. eLife. 27, e32696. 10.7554/eLife.32696 29911970 PMC6039182

[B40] Kelley W. M. Macrae C. N. Wyland C. L. Caglar S. Inati S. Heatherton T. F. (2002). Finding the self? An event-related fMRI study. *J. Cogn*. Neurosci. 14, 785–794. 10.1162/08989290260138672 12167262

[B41] Kragel P. A. Knodt A. R. Hariri A. R. LaBar K. S. (2016). Decoding spontaneous emotional states in the human brain. PLoS Biol. 14, e2000106. 10.1371/journal.pbio.2000106 27627738 PMC5023171

[B42] Kucyi A. Hove M. J. Esterman M. Hutchison R. M. Valera E. M. (2017). Dynamic brain network correlates of spontaneous fluctuations in attention. *Cereb*. Cortex 27, 1831–1840. 10.1093/cercor/bhw029 26874182 PMC6317462

[B43] Laumann T. O. Snyder A. Z. Mitra A. Gordon E. M. Gratton C. Adeyemo B.. (2017). On the stability of BOLD fMRI correlations. *Cereb*. Cortex 27, 4719–4732. 10.1093/cercor/bhw265 27591147 PMC6248456

[B44] Leonardi N. Richiardi J. Gschwind M. Simioni S. Annoni J. M. Schluep M.. (2013). Principal components of functional connectivity: A new approach to study dynamic brain connectivity during rest. NeuroImage 83, 937–950. 10.1016/j.neuroimage.2013.07.019 23872496

[B45] Leonardi N. Van De Ville D. (2015). On spurious and real fluctuations of dynamic functional connectivity during rest. NeuroImage 104, 430–436. 10.1016/j.neuroimage.2014.09.007 25234118

[B46] Liegeois R. Laumann T. O. Snyder A. Z. Zhou J. Yeo B. T. T. (2017). Interpreting temporal fluctuations in resting-state functional connectivity MRI. NeuroImage 163, 437–455. 10.1016/j.neuroimage.2017.09.012 28916180

[B47] Lindquist M. A. Xu Y. Nebel M. B. Caffo B. S. (2014). Evaluating dynamic bivariate correlations in resting-state fMRI: A comparison study and a new approach. NeuroImage 101, 531–546. 10.1016/j.neuroimage.2014.06.052 24993894 PMC4165690

[B48] Liu X. Duyn J. H. (2013). Time-varying functional network information extracted from brief instances of spontaneous brain activity. *Proc. Natl. Acad*. Sci. USA. 110, 4392–4397. 10.1073/pnas.1216856110 23440216 PMC3600481

[B49] Ma Y. Shaik M. A. Kozberg M. G. Kim S. H. Portes J. P. Timerman D.. (2016). Resting-state hemodynamics are spatiotemporally coupled to synchronized and symmetric neural activity in excitatory neurons. Proc. Natl. Acad. Sci. USA. 113, E8463–E8471. 10.1073/pnas.1525369113 27974609 PMC5206542

[B50] Majeed W. Magnuson M. Hasenkamp W. Schwarb H. Schumacher E. H. Barsalou L.. (2011). Spatiotemporal dynamics of low frequency BOLD fluctuation in rats and humans. NeuroImage 54, 1140–1150. 10.1016/j.neuroimage.2010.08.030 20728554 PMC2997178

[B51] Marcus D. S. Harms M. P. Snyder A. Z. Jenkinson M. Wilson J. A. Glasser M. F.. (2013). Human Connectome Project informatics: quality control, database services, and data visualization. NeuroImage 80, 202–219. 10.1016/j.neuroimage.2013.05.077 23707591 PMC3845379

[B52] Margulies D. S. Ghosh S. S. Goulas A. Falkiewicz M. Huntenburg J. M. Langs G.. (2016). Situating the default-mode network along a principal gradient of macroscale cortical organization. Proc. Natl. Acad. Sci. USA. 113, 12574–12579. 10.1073/pnas.1608282113 27791099 PMC5098630

[B53] Matsui T. Hattori Y. Tsumura K. Aoki R. Takeda M. Nakahara K.. (2022b). Executive control by fronto-parietal activity explains counterintuitive decision behavior in complex value-based decision-making. NeuroImage 249, 118892. 10.1016/j.neuroimage.2022.118892 35007716

[B54] Matsui T. Murakami T. Ohki K. (2016). Transient neuronal coactivations embedded in globally propagating waves underlie resting-state functional connectivity. Proc. Natl. Acad. Sci. USA. 113, 6556–6561. 10.1073/pnas.1521299113 27185944 PMC4988587

[B55] Matsui T. Murakami T. Ohki K. (2018). Mouse optical imaging for understanding resting-state functional connectivity in human fMRI. Commun. Integr. Biol. 11, e1528821. 10.1080/19420889.2018.1528821 30534348 PMC6284571

[B56] Matsui T. Murakami T. Ohki K. (2019). Neuronal origin of the temporal dynamics of spontaneous BOLD activity correlation. *Cereb*. Cortex 29, 1496–1508. 10.1093/cercor/bhy045 29522092

[B57] Matsui T. Pham T. Q. Jimura K. Chikazoe J. (2022a). On co-activation pattern analysis and non-stationarity of resting brain activity. NeuroImage 249, 118904. 10.1016/j.neuroimage.2022.118904 35031473

[B58] Miller K. L. Alfaro-Almagro F. Bangerter N. K. Thomas D. L. Yacoub E. Xu J. Q.. (2016). Multimodal population brain imaging in the UK Biobank prospective epidemiological study. *Nat*. Neurosci. 19, 1523–1536. 10.1038/nn.4393 27643430 PMC5086094

[B59] Mitchell J. P. Banaji M. R. Macrae C. N. (2005). General and specific contributions of the medial prefrontal cortex to knowledge about mental states. NeuroImage 28, 757–762. 10.1016/j.neuroimage.2005.03.011 16325141

[B60] Mitra A. Snyder A. Z. Blazey T. Raichle M. E. (2015). Lag threads organize the brain's intrinsic activity. Proc. Natl. Acad. Sci. USA 112, E2235–E2244. 10.1073/pnas.1503960112 25825720 PMC4418865

[B61] Nomi J. S. Vij S. G. Dajani D. R. Steimke R. Damaraju E. Rachakonda S.. (2017). Chronnectomic patterns and neural flexibility underlie executive function. NeuroImage 147, 861–871. 10.1016/j.neuroimage.2016.10.026 27777174 PMC5303676

[B62] Piefke M. Weiss P. H. Zilles K. Markowitsch H. J. Fink G. R. (2003). Differential remoteness and emotional tone modulate the neural correlates of autobiographical memory. Brain 126, 650–668. 10.1093/brain/awg064 12566286

[B63] Ponce-Alvarez A. Deco G. Hagmann P. Romani G. L. Mantini D. Corbetta M. (2015). Resting-state temporal synchronization networks emerge from connectivity topology and heterogeneity. PLoS Comp. Biol. 11, e1004100. 10.1371/journal.pcbi.1004100 25692996 PMC4333573

[B64] Power J. D. Barnes K. A. Snyder A. Z. Schlaggar B. L. Petersen S. E. (2012). Spurious but systematic correlations in functional connectivity MRI networks arise from subject motion. NeuroImage 59, 2142–2154. 10.1016/j.neuroimage.2011.10.018 22019881 PMC3254728

[B65] Power J. D. Cohen A. L. Nelson S. M. Wig G. S. Barnes K. A. Church J. A.. (2011). Functional network organization of the human brain. Neuron 72, 665–678. 10.1016/j.neuron.2011.09.006 22099467 PMC3222858

[B66] Power J. D. Mitra A. Laumann T. O. Snyder A. Z. Schlaggar B. L. Petersen S. E. (2014). Methods to detect, characterize, and remove motion artifact in resting state fMRI. NeuroImage 84, 320–341. 10.1016/j.neuroimage.2013.08.048 23994314 PMC3849338

[B67] Power J. D. Plitt M. Laumann T. O. Martin A. (2017). Sources and implications of whole-brain fMRI signals in humans. NeuroImage 146, 609–625. 10.1016/j.neuroimage.2016.09.038 27751941 PMC5321814

[B68] Raichle M. E. MacLeod A. M. Snyder A. Z. Powers W. J. Gusnard D. A. Shulman G. L. (2001). A default mode of brain function. Proc. Natl. Acad. Sci. USA. 98, 676–682. 10.1073/pnas.98.2.676 11209064 PMC14647

[B69] Rashid B. Damaraju E. Pearlson G. D. Calhoun V. D. (2014). Dynamic connectivity states estimated from resting fMRI Identify differences among Schizophrenia, bipolar disorder, and healthy control subjects. Front. Hum. Neurosci. 8, 897. 10.3389/fnhum.2014.00897 25426048 PMC4224100

[B70] Rushworth M. F. S. Walton M. E. Kennerley S. W. Bannerman D. M. (2004). Action sets and decisions in the medial frontal cortex. Trends. Cogn. Sci. 8, 410–417. 10.1016/j.tics.2004.07.009 15350242

[B71] Sakoglu U. Pearlson G. D. Kiehl K. A. Wang Y. M. Michael A. M. Calhoun V. D. (2010). A method for evaluating dynamic functional network connectivity and task-modulation: application to schizophrenia. Magn. Res. Mat. Phys. Biol. Med. 23, 351–366. 10.1007/s10334-010-0197-8 20162320 PMC2891285

[B72] Salimi-Khorshidi G. Douaud G. Beckmann C. F. Glasser M. F. Griffanti L. Smith S. M. (2014). Automatic denoising of functional MM data: Combining independent component analysis and hierarchical fusion of classifiers. NeuroImage 90, 449–468. 10.1016/j.neuroimage.2013.11.046 24389422 PMC4019210

[B73] Senden M. Reuter N. van den Heuvel M. P. Goebel R. Deco G. (2017). Cortical rich club regions can organize state-dependent functional network formation by engaging in oscillatory behavior. NeuroImage 146, 561–574. 10.1016/j.neuroimage.2016.10.044 27989843

[B74] Shine J. M. Bissett P. G. Bell P. T. Koyejo O. Balsters J. H. Gorgolewski K. J.. (2016a). The dynamics of functional brain networks: Integrated network states during cognitive task performance. Neuron 92, 544–554. 10.1016/j.neuron.2016.09.018 27693256 PMC5073034

[B75] Shine J. M. Koyejo O. Poldrack R. A. (2016b). Temporal metastates are associated with differential patterns of time-resolved connectivity, network topology, and attention. Proc. Natl. Acad. Sci. USA. 113, 9888–9891. 10.1073/pnas.1604898113 27528672 PMC5024627

[B76] Smith S. M. Miller K. L. Moeller S. Xu J. Auerbach E. J. Woolrich M. W.. (2012). Temporally-independent functional modes of spontaneous brain activity. Proc. Natl. Acad. Sci. USA 109, 3131–3136. 10.1073/pnas.1121329109 22323591 PMC3286957

[B77] Smith S. M. Nichols T. E. Vidaurre D. Winkler A. M. Behrens T. E. J. Glasser M. F.. (2015). A positive-negative mode of population covariation links brain connectivity, demographics and behavior. *Nat*. Neurosci. 18, 1565–1567. 10.1038/nn.4125 26414616 PMC4625579

[B78] Solovey G. Alonso L. M. Yanagawa T. Fujii N. Magnasco M. O. Cecchi G. A.. (2015). Loss of consciousness is associated with stabilization of cortical activity. J. Neurosci. 35, 10866–10877. 10.1523/JNEUROSCI.4895-14.2015 26224868 PMC4518057

[B79] Stoffers D. Diaz B. A. Chen G. den Braber A. van 't Ent D. Boomsma D. I.. (2015). Resting-state fMRI functional connectivity is associated with sleepiness, imagery, and discontinuity of mind. PLoS ONE 10, e0142014. 10.1371/journal.pone.0142014 26540239 PMC4634926

[B80] Su J. P. Shen H. Zeng L. L. Qin J. Liu Z. N. Hu D. W. (2016). Heredity characteristics of schizophrenia shown by dynamic functional connectivity analysis of resting-state functional MRI scans of unaffected siblings. Neuroreport 27, 843–848. 10.1097/WNR.0000000000000622 27295028

[B81] Summerfield J. J. Hassabis D. Maguire E. A. (2009). Cortical midline involvement in autobiographical memory. NeuroImage 44, 1188–1200. 10.1016/j.neuroimage.2008.09.033 18973817 PMC2625448

[B82] Tagliazucchi E. Balenzuela P. Fraiman D. Chialvo D. R. (2012). Criticality in large-scale brain fMRI dynamics unveiled by a novel point process analysis. *Front*. Physiol. 3, 15. 10.3389/fphys.2012.00015 22347863 PMC3274757

[B83] Tagliazucchi E. Laufs H. (2014). Decoding wakefulness levels from typical fMRI resting-State data reveals reliable drifts between wakefulness and sleep. Neuron 82, 695–708. 10.1016/j.neuron.2014.03.020 24811386

[B84] Van der Waerden B. L. (1952). On the method of saddle points. Appl. Sci. Res. B 2, 33–45. 10.1007/BF02919754

[B85] Vergara V. M. Mayer A. R. Damaraju E. Hutchison K. Calhoun V. D. (2017). The effect of preprocessing pipelines in subject classification and detection of abnormal resting state functional network connectivity using group ICA. Neuroimage 145, 365–376. 10.1016/j.neuroimage.2016.03.038 27033684 PMC5035165

[B86] Vidaurre D. Smith S. M. Woolrich M. W. (2017). Brain network dynamics are hierarchically organized in time. Proc. Natl. Acad. Sci. USA. 114, 12827–12832. 10.1073/pnas.1705120114 29087305 PMC5715736

[B87] Vohryzek J. Deco G. Cessac B. Kringelbach M. L. Cabral J. (2020). Ghost attractors in spontaneous brain activity: Recurrent excursions into functionally-relevant BOLD phase-locking states. Front. Syst. Neurosci. 14, 20. 10.3389/fnsys.2020.00020 32362815 PMC7182014

[B88] Winder A. T. Echagarruga C. Zhang Q. Drew P. J. (2017). Weak correlations between hemodynamic signals and ongoing neural activity during the resting state. Nat. Neurosci. 20, 1761–1769. 10.1038/s41593-017-0007-y 29184204 PMC5816345

[B89] Xia M. Wang J. He Y. (2013). BrainNet viewer: a network visualization tool for human brain connectomics. PLoS ONE 8, e68910. 10.1371/journal.pone.0068910 23861951 PMC3701683

[B90] Yarkoni T. Poldrack R. A. Nichols T. E. van Essen D. C. Wager T. D. (2011). Large-scale automated synthesis of human functional neuroimaging data. *Nat*. Methods 8, 665–670. 10.1038/nmeth.1635 21706013 PMC3146590

[B91] Zalesky A. Breakspear M. (2015). Towards a statistical test for functional connectivity dynamics. NeuroImage 114, 466–470. 10.1016/j.neuroimage.2015.03.047 25818688

[B92] Zalesky A. Fornito A. Cocchi L. Gollo L. L. Breakspear M. (2014). Time-resolved resting-state brain networks. Proc. Natl. Acad. Sci. USA. 111, 10341–10346. 10.1073/pnas.1400181111 24982140 PMC4104861

