# Supplementary material for: A method for reconstruction of interpretable brain networks from transient synchronization in resting-state BOLD fluctuations
Source: Front Neuroinform. 2023 Jan 12;16:960607. doi: 10.3389/fninf.2022.960607 (PMC9878402; doi:10.3389/fninf.2022.960607)
Supplement: Supplementary file 1 [file Data_Sheet_1.pdf]

Table S1. Summary of clusters identified in the current study.

Cluster	Share	Regions	Related figures	Putative interpretation	Reference
**1**	23.4%	Visual sensory, FPN / DMN	Fig. 5	General task processing / Mind wondering	Dosenbach et al., 2006, 2008.
**2**	16.6%	Visual, FPN, salience / DMN	Figs. 3, 4B, 5, 6	Cognitive or visual mental imagery	Allen et al., 2014 Dosenbach et al., 2006, 2008.
**3**	13.3%	Cingulo-opercular / DMN, visual	Fig. 5	Heightened somatosensory sensations	N/A
**4**	15.9%	Visual, sensory	Figs. 4B, 5B	Attention evoked and/or head motion	N/A
**5**	13.0%	FPN / DMN, visual	Figs. 4B, 5	Mind wondering	N/A
**6**	18.5%	Visual, sensory, DMN	Figs. 3, 4, 7	Drowsiness / Internal language processing	Allen et al., 2014; Taguriazucchi & Laufs 2014.

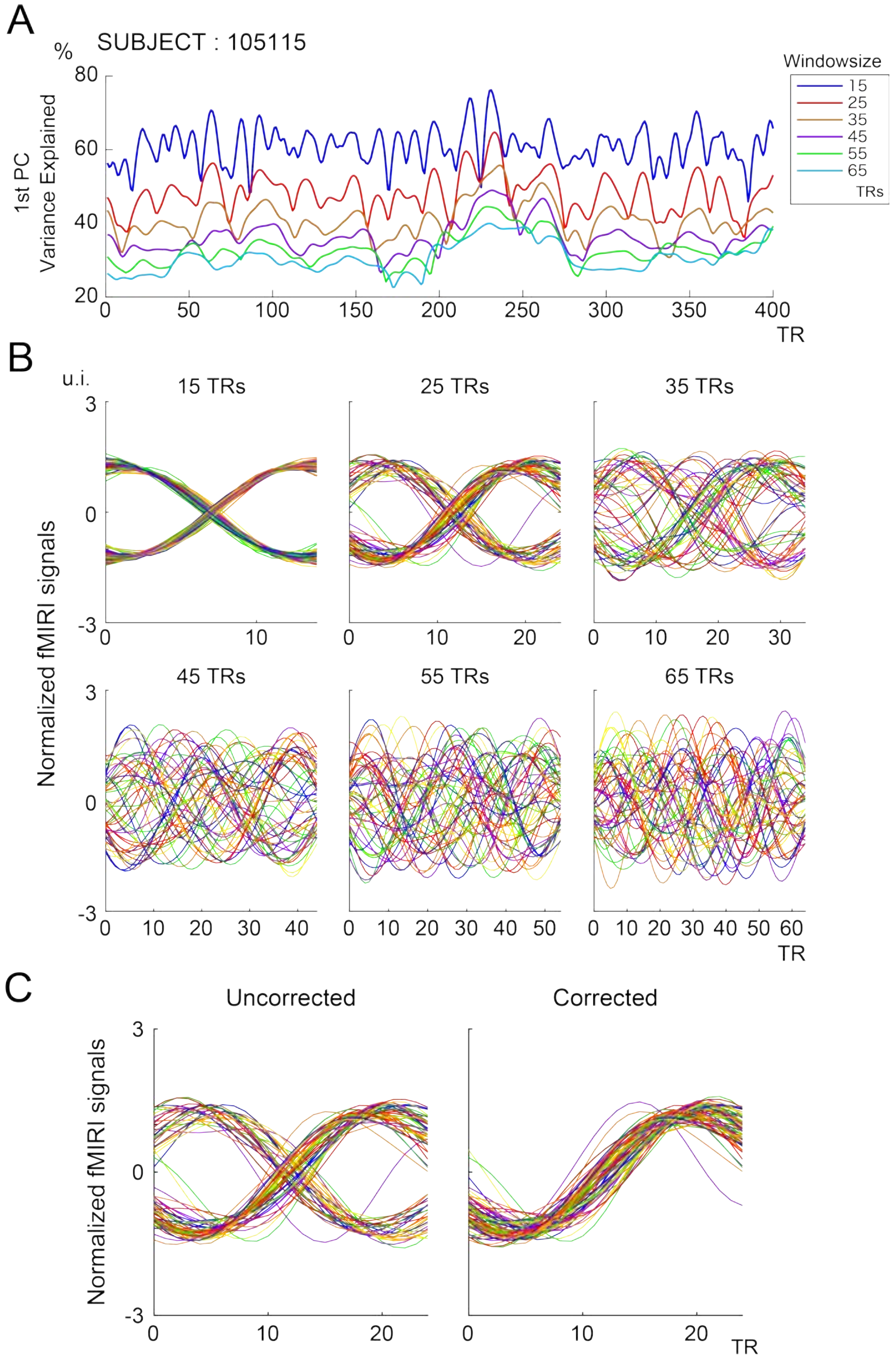

Figure S1. A) Exemplar timecourses of variance explained by the 1st PC along scanning frames for one participant (ID: 105115). Line colors indicate the sliding window size as shown on the *right*. The current study used 25 TRs in the subsequent analyses (red). B) MRI signal timecourses in ROIs for windows corresponding to TS for the same participant. Each line indicates the MRI signal of the 1st PC across ROIs where the variance explained by the 1st PC showed a local maximum (i.e., TS). Horizontal and vertical axes indicate relative time points in the window and fMRI signals, and the center of the horizontal axis represents TS. Each of the 6 panels shows the signal timecourse for one window size as shown on the top of the panel. C) Due to the arbitrariness of the sign of the 1st PC, MRI signal timecourses of the 1st PC were corrected by flipping the sign of the 1st PC such that the MRI signals monotonically increase around TS.

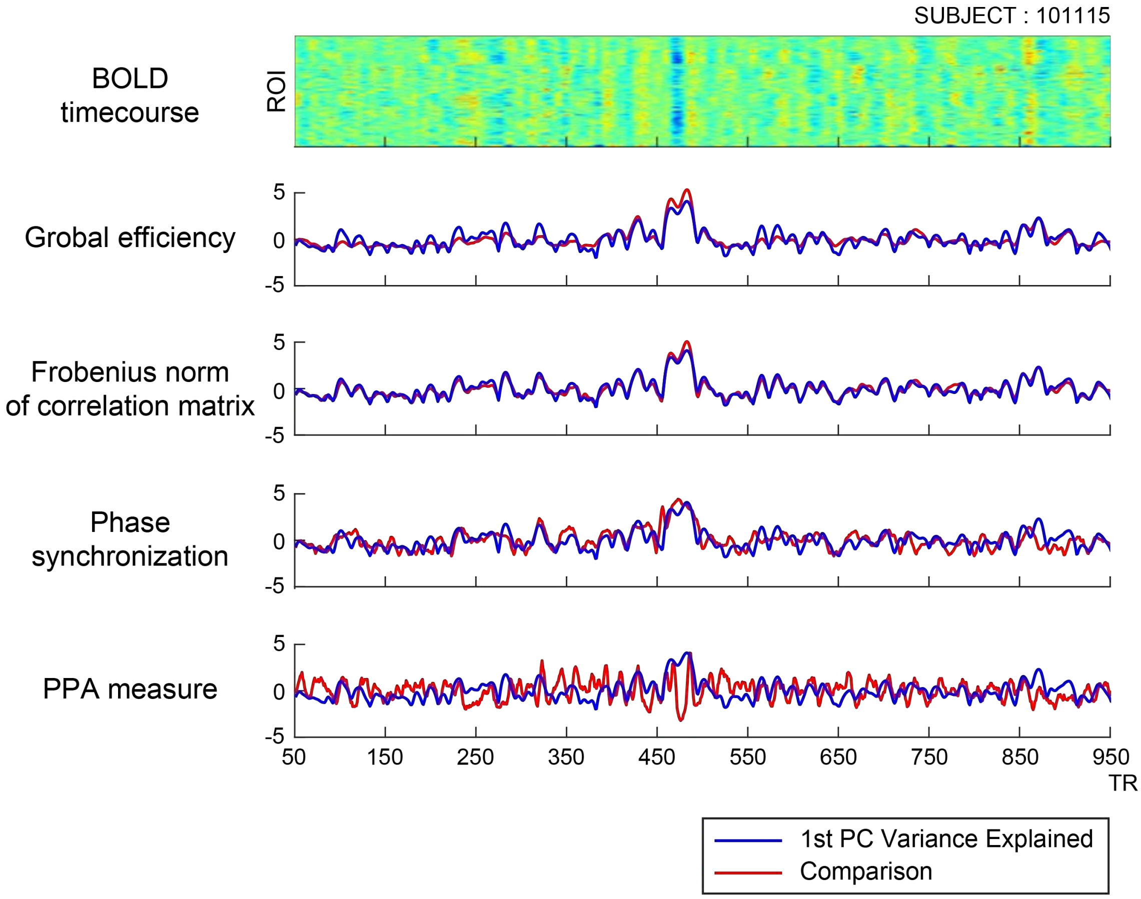

Figure S2. TS and relevant measures of correlations. Timecourses of MRI signal in all ROIs (*top*). The four panels indicate a comparison of TS (blue line) and comparable measures (red line) as indicated on the left.

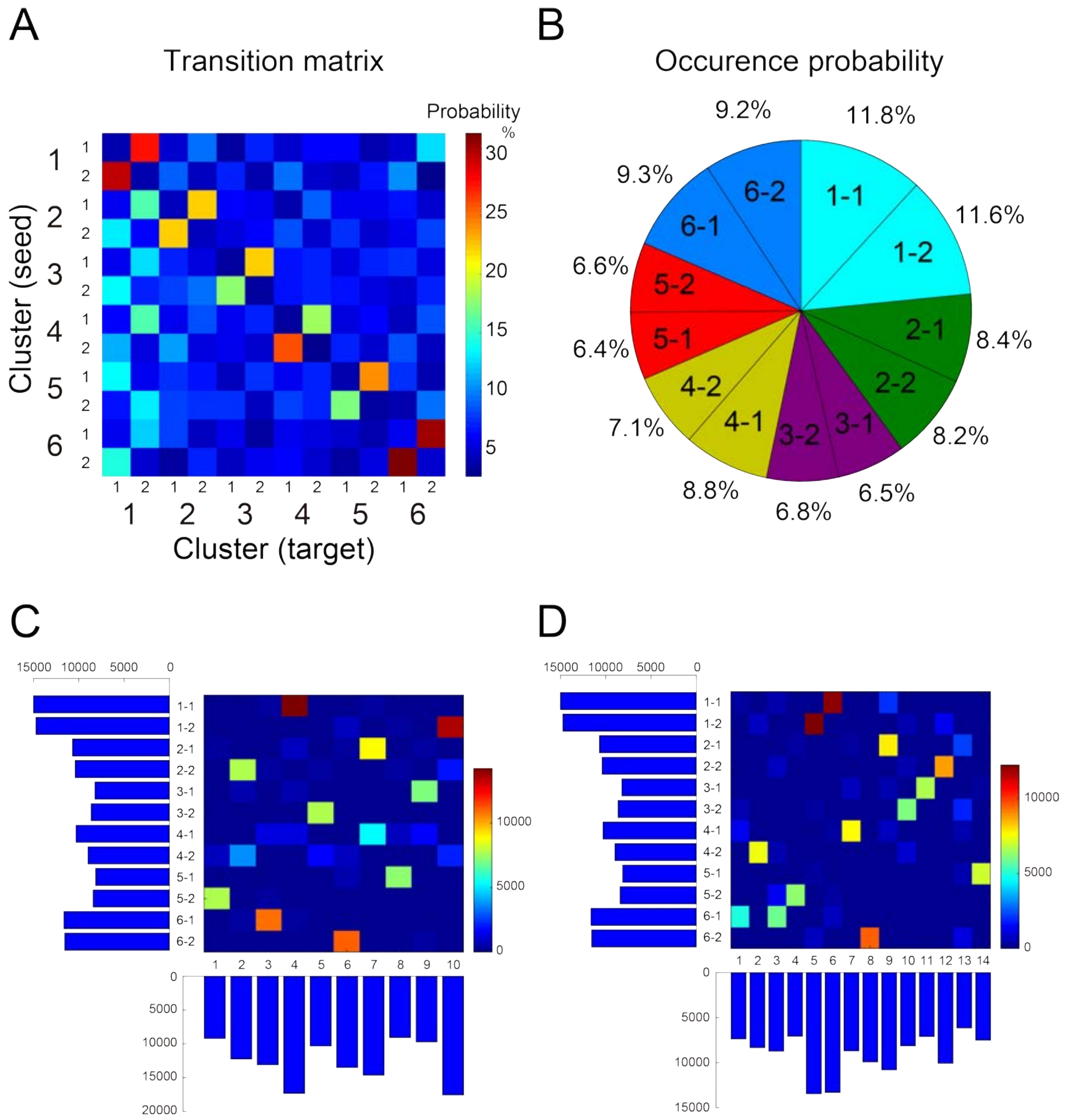

Figure S3. Transition pattern of TSP. A) Transition matrix of TSPs. The row and column indicate target and seed clusters, respectively. Transition probabilities are color-coded and mapped on the heatmap. B) Occurrence probability of the clusters. The colors of the pie chart indicate cluster pairs, and the probability is indicated outside the chart. C, D) Clustering was performed by changing the number of clusters (C: 10 clusters; D: 14 clusters), and then each TSP occurrence was matched to the cluster within the 12 (sub-)clusters as indicated in panels A, B) based on the time of the occurrence. The frequency of the matched cluster occurrence is color-coded in 2 × 2 heatmaps. The column indicates the 12 clusters and row indicates the 10 (D) and 14 (C) clusters. The bar diagrams on the *left* and *bottom* show the total occurrence frequency of each cluster.

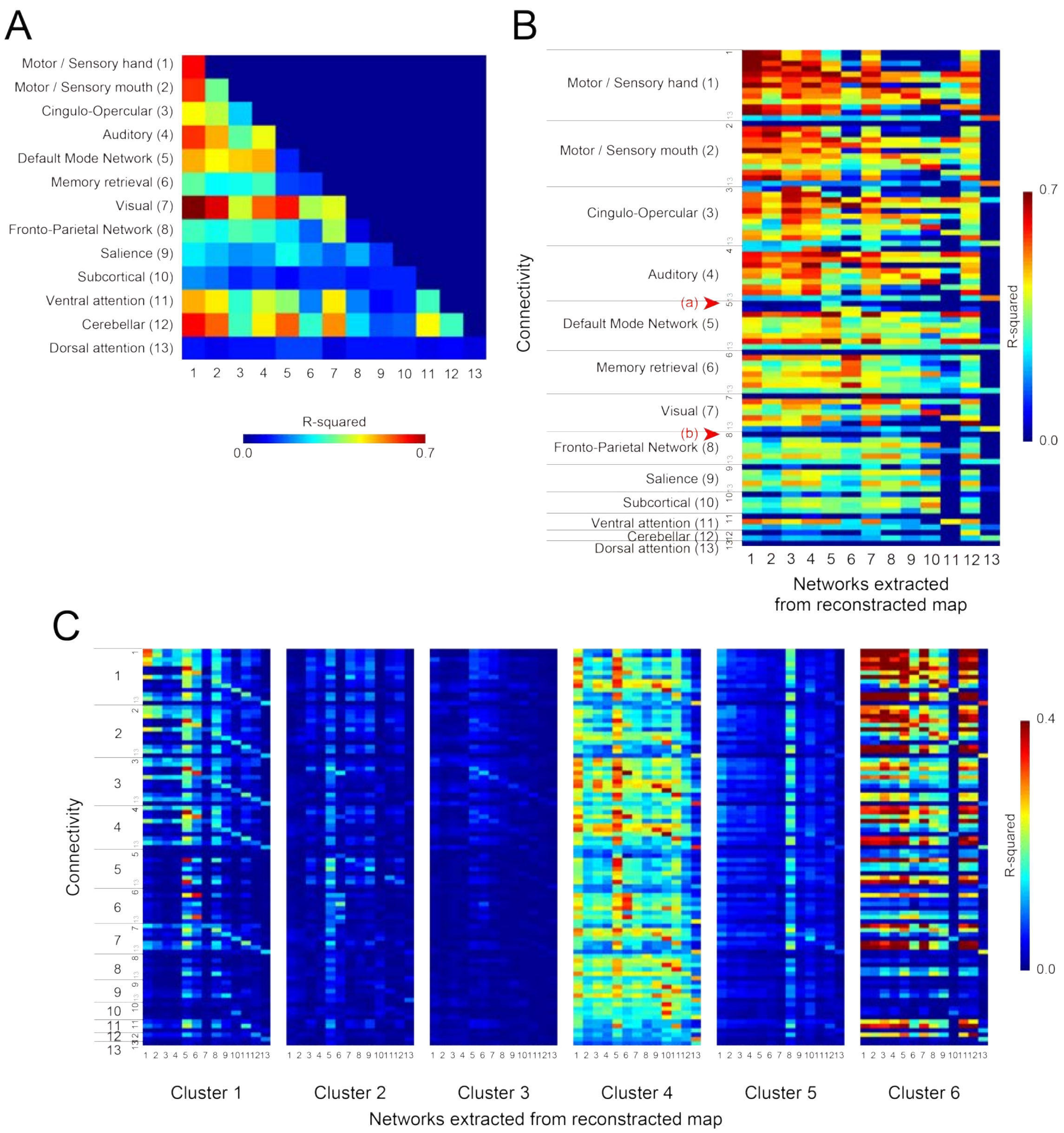

Figure S4. Relationships between RSFC and TSP. A) The strength of pairwise RSFC networks was predicted by the occurrence of clusters. R-squared values are color-coded in the heatmap for each pair of RSFC. The numbers indicate RSFC networks. B) The strength of the pairwise RSFC was predicted by reconstruction maps of the 6 clusters (Fig. 2) based on a multiple regression model, and R-squared values are color-coded in the heatmap. The formats are similar to those in Panel A. Red arrowheads indicate DMN (a) and FPN (b). C) The strength of the pairwise RSFC was predicted by clusters based on a simple regression analysis. R-squared values are color-coded, and heatmaps were created for each cluster as indicated at the bottom. Formats are similar to those in Panels A, B.

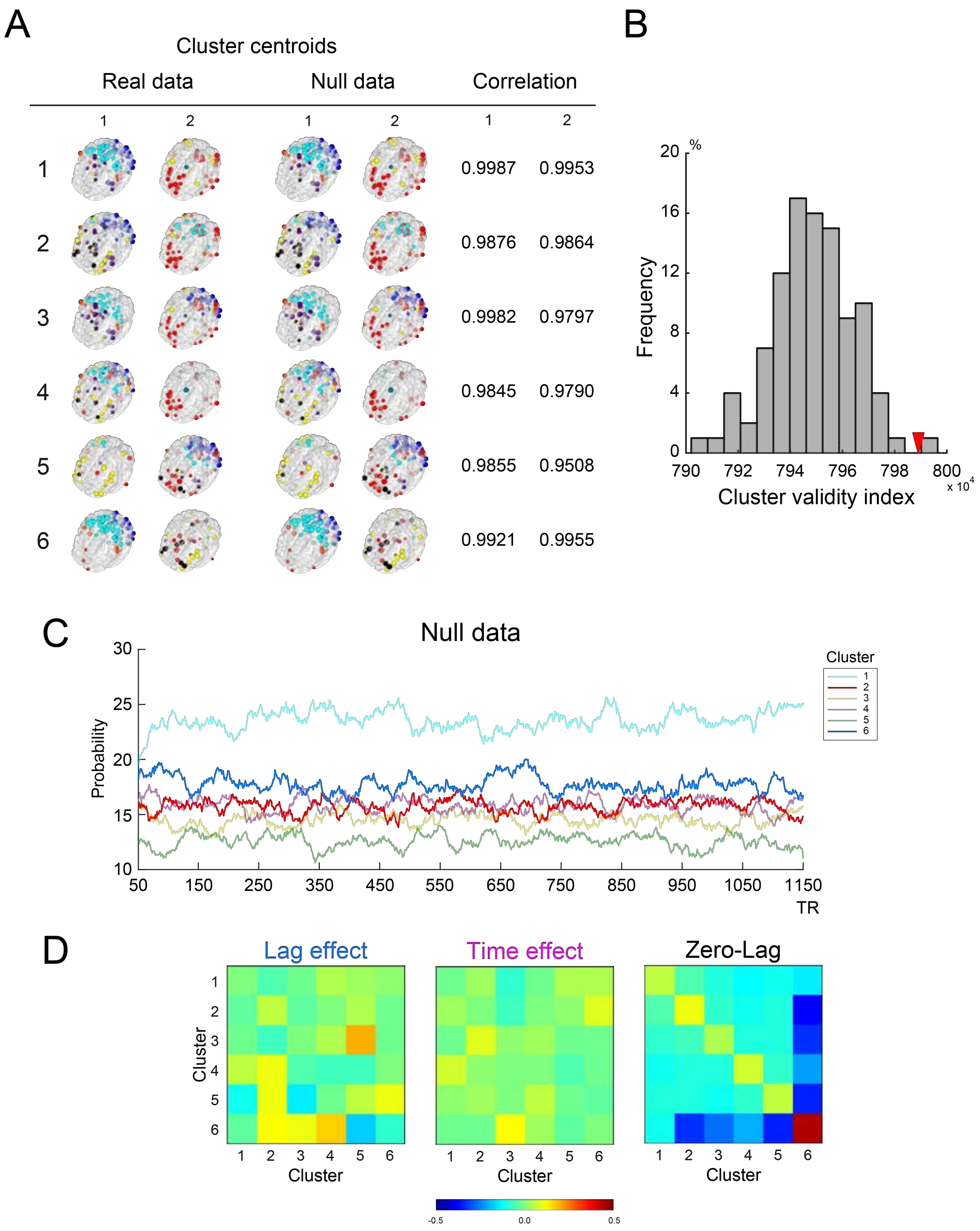

Figure S5. Analysis of temporal characteristics based on phase-randomized null data that preserved the autocorrelation of the real data. A) Cluster centroids were compared between those created based on real data (*left*) and null data (*middle*). Correlation between centroids between real and null data (*right*). B) Histogram of cluster validity index calculated based on real data (red arrowhead) and 100 sets of null data (gray). C) Sample timecourses of the null data. Colors indicate the clusters as shown on the *right*. D) Based on the null data, PMI matrices predicted by lag effect, time effect, and zero-lag effect and regression coefficients for all cluster combinations are color-coded for each effect.

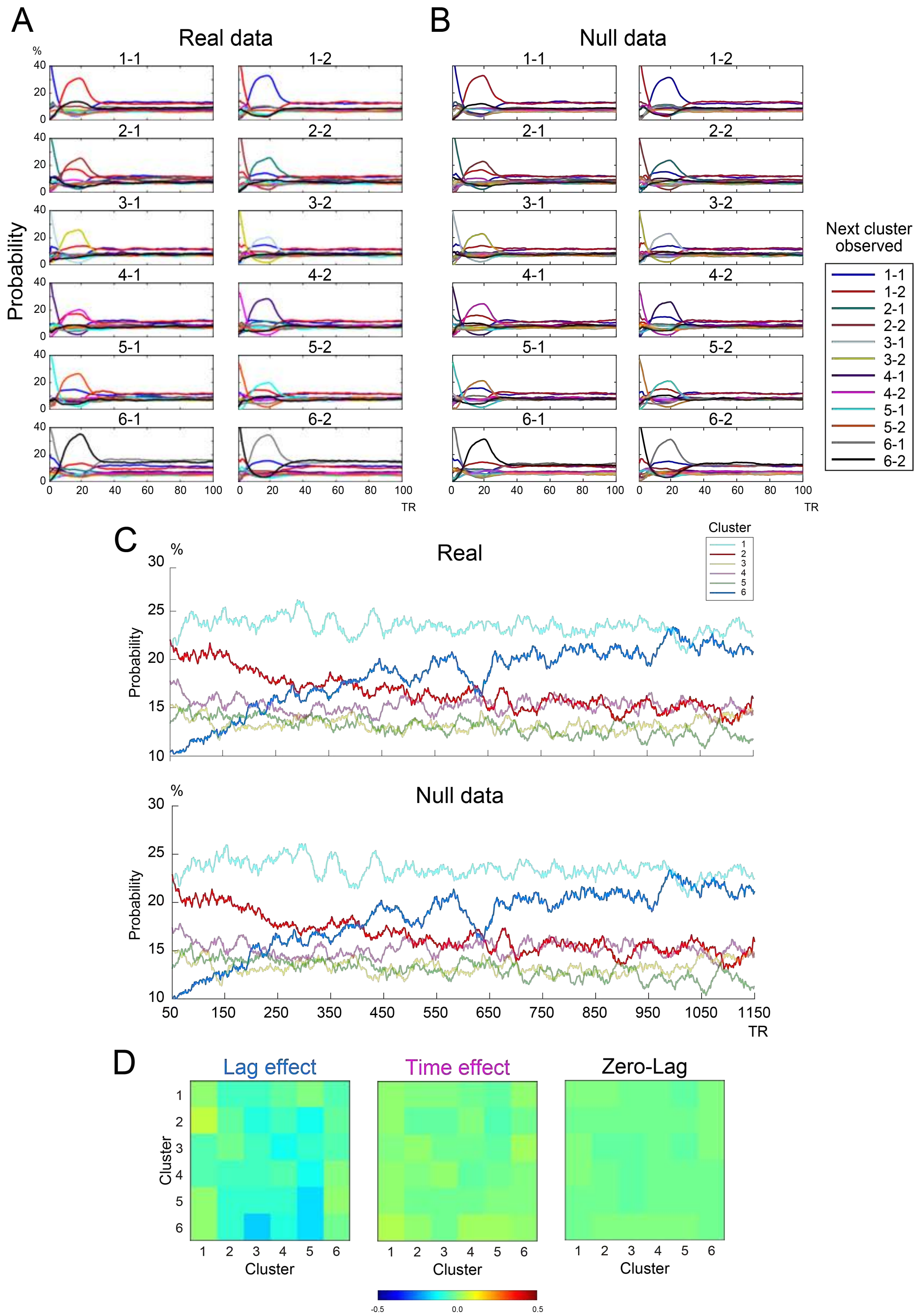

Figure S6. Analysis of temporal characteristics based on phase-randomized null data that preserved the temporal characteristics of cluster occurrence probability of the real data. A, B) Timecourse of the conditional probability of cluster occurrence given the occurrence of another cluster. Horizontal and vertical axes show time and probability, respectively. Each panel indicates one cluster. A) Real data. B) Null data. The cluster occurrence probability of the real data (A) was preserved in the null data (B). Sample timecourses of the null data. *Top*: real data; *bottom*: null data. Colors indicate the clusters as shown on the *right*. D) Based on the null data, PMI matrices predicted by lag effect, time effect, and zero-lag effect and regression coefficients for all cluster combinations are color-coded for each effect.

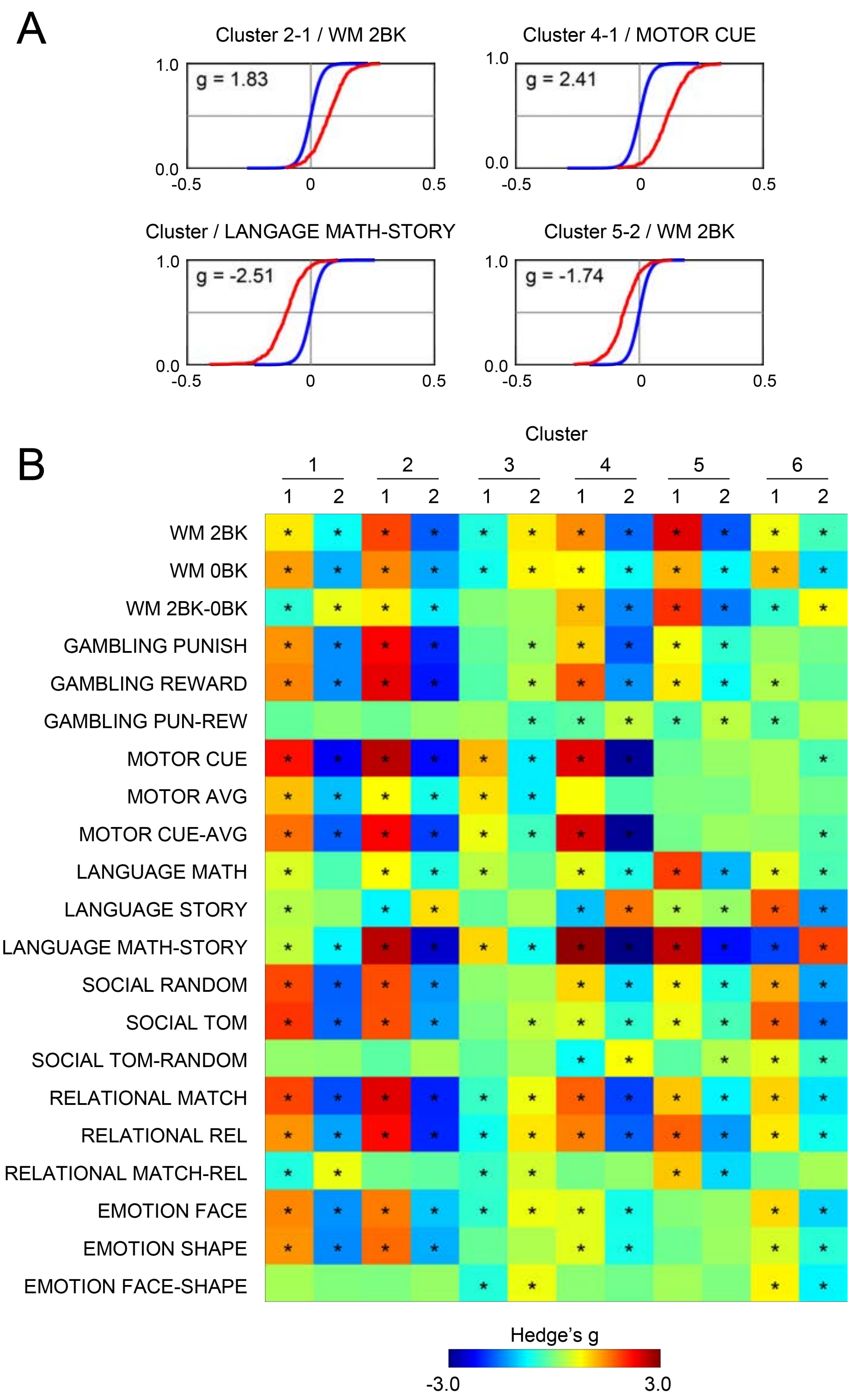

Figure S7. A) Sample cumulative distribution of spatial correlation between cluster reconstruction maps and task activation maps. Red and blue lines indicate cumulative distributions calculated based on real data and null data. Correlation is shown at the top of each panel. The effect size of the correlation is shown on top left of each panel as Hedges' g. B) Hedges' g between each of the 12 reconstructed maps and task-related activation. *: $P < 0.05$ uncorrected.
